# Supplementary material for: Phase I study on the pharmacokinetics of intravaginal, self-administered artesunate vaginal pessaries among women in Kenya
Source: PLoS One. 2025 Apr 9;20(4):e0316334. doi: 10.1371/journal.pone.0316334 (PMC11981651; doi:10.1371/journal.pone.0316334)
Supplement: S2 File — (DOCX) [file pone.0316334.s002.docx]

**IGHID 12333: Phase I study on the Pharmacokinetics of intravaginal, self-administered Artesunate vaginal pessaries among women in Kenya.**

**Principal Investigator**

Chemtai Mungo, MD, MPH

University of North Carolina Chapel Hill School of Medicine

**Co-Principal Investigator**

Jackton Omoto, MBChB, MMed

Maseno University School of Medicine =

**Co-Investigator**

Cirillus Ogollah, BSc, DipCM, Kenya Medical Research Institute

**Funding Sponsor**: University of North Carolina-Chapel Hill, Department of Obstetrics and Gynecology

**Version Date:** October 18, 2024

**Version Number**: 4.0

Table of Contents

[**I. List of Abbreviations 4**](#_heading=h.gjdgxs)

[**II. Study Abstract/Synopsis 5**](#_heading=h.30j0zll)

[**III. Protocol Summary 6**](#_heading=h.1fob9te)

[**IV. Lay Summary 7**](#_heading=h.3znysh7)

[**1.0 LITERATURE REVIEW 8**](#_heading=h.2et92p0)

[1.1 Intravaginal Artesunate for Cervical Precancer Treatment 8](#_heading=h.tyjcwt)

[1.1.1 Preclinical Studies 8](#_heading=h.3dy6vkm)

[1.1.1.1 Mechanism of action 8](#_heading=h.1t3h5sf)

[1.1.2 Clinical Studies 9](#_heading=h.4d34og8)

[1.1.2.1 Safety and Toxicity for Intravaginal Use 9](#_heading=h.2s8eyo1)

[1.2 Summary of Artesunate Pharmacokinetics 9](#_heading=h.17dp8vu)

[1.3 Effect of malaria infection status on artesunate and DHA pharmacokinetics 11](#_heading=h.3rdcrjn)

[1.4 Malaria treatment and artemisinin resistance 11](#_heading=h.26in1rg)

[1.5 Systemic Artesunate absorption and possible implications for developing resistance for malaria treatment. 13](#_heading=h.lnxbz9)

[**2.0 BACKGROUND AND RATIONALE 13**](#_heading=h.35nkun2)

[2.1 Background 13](#_heading=h.1ksv4uv)

[**2.2 Problem Statement and Study Rationale (Justification) 14**](#_heading=h.44sinio)

[**3.0 RESEARCH HYPOTHESIS AND OBJECTIVES 14**](#_heading=h.2jxsxqh)

[3.1 Research Hypothesis 14](#_heading=h.z337ya)

[3.2 Research Objectives 14](#_heading=h.3j2qqm3)

[3.3 Conceptual Framework 15](#_heading=h.1y810tw)

[3.4 Study Schema 16](#_heading=h.4i7ojhp)

[3.5 Rationale for Study Design 16](#_heading=h.2xcytpi)

[**4.0 STUDY DRUG AND ADMINISTRATION 18**](#_heading=h.1ci93xb)

[4.1. Description of the Study Drug 18](#_heading=h.3whwml4)

[4.2 Dosage and Administration 19](#_heading=h.2bn6wsx)

[4.3 Accountability Procedures for Study Investigational Drug 20](#_heading=h.3as4poj)

[**5.0 METHODOLOGY 20**](#_heading=h.1pxezwc)

[5.1 Study Details and Procedures 20](#_heading=h.49x2ik5)

[5.2 Study Activities by Visit 22](#_heading=h.2p2csry)

[5.3 Adverse Event Reporting 24](#_heading=h.147n2zr)

[5.4 Specimen collection, processing, storage and analysis 27](#_heading=h.3o7alnk)

[5.5 Data Collection and Management 30](#_heading=h.32hioqz)

[5.6 Statistical Considerations and Data analysis 31](#_heading=h.1hmsyys)

[5.6.1 Sample Size: 31](#_heading=h.41mghml)

[5.6.2 Data Analysis Plan 32](#_heading=h.2grqrue)

[5.7 Community Engagement Plan 34](#_heading=h.vx1227)

[5.8 Study Limitations, Risks and Mitigation of Risks 34](#_heading=h.3fwokq0)

[5.9 Plans for Communicating Study Findings 35](#_heading=h.1v1yuxt)

[**6.0 ETHICAL CONSIDERATIONS 35**](#_heading=h.4f1mdlm)

[6.1 Institutional Review Board (IRB) Approval and Consent 35](#_heading=h.3tbugp1)

[6.2 Confidentiality and Privacy 36](#_heading=h.206ipza)

[6.3 Potential Benefits 36](#_heading=h.4k668n3)

[6.4 Potential Harms and Minimization of Harms 36](#_heading=h.2zbgiuw)

[**7.0 STUDY MANAGEMENT AND ORGANIZATION 37**](#_heading=h.1egqt2p)

[7.1 Study Management 37](#_heading=h.3ygebqi)

[7.2 Role of Investigators Principal Investigators and role of Principal Co-Investigators 39](#_heading=h.2dlolyb)

[7.3 Study Timeline 40](#_heading=h.sqyw64)

[7.4 Budget and Justification 40](#_heading=h.3cqmetx)

[**8.0 REFERENCES 41**](#_heading=h.1rvwp1q)

[**9.0 Appendices 46**](#_heading=h.4bvk7pj)

## **List of Abbreviations**

AE Adverse Events

API Active Pharmaceutical Ingredient

AUC Area under the curve

AS Artesunate

CIN2/3 Cervical Intraepithelial Neoplasia grade 2 and 3

CPO Clinical Protocol Office

DSMC Data and Safety and Monitoring Committee

DHA Dihydroartemisinin

ESRC Ethics & Scientific Review Committee

GCP Good Clinical Practice

HIV Human Immunodeficiency Virus

HPV Human papillomavirus

IRB Institutional Review Board

KEMRI Kenya Medical Research Institute

LCCC Lineberger Comprehensive Cancer Center

LEEP Loop Electrosurgical Excision Procedure

LMIC Low- and middle-Income countries

NCI-CTCAE National Cancer Institute Common Terminology Criteria for Adverse Events

PI Principal Investigator

PK Pharmacokinetics

PPB Pharmacy and Poisons Board

NACOSTI National Commission for Science, Technology & Innovation

STI Sexually Transmitted Infections

SOP Standard Operating Procedure

WHO World Health Organization

WLWH Women Living with HIV

UNC University of North Carolina

## **Study Abstract/Synopsis**

**Background:** While preventable, cervical cancer is the second most common cancer among women globally, accounting for approximately 600,000 new cases and 300,000 deaths per year. Due to lack of access to primary and secondary prevention, women living in low-and middle-income countries bear a disproportionate burden of cervical cancer, accounting for 90% of new cases and 85% of deaths globally. Cervical cancer can be prevented through vaccination against Human papillomavirus (HPV), whose infection is required to develop cervical cancer. Among unvaccinated women, screening for HPV or cervical precancer allows identification of precancerous lesions – primarily cervical intraepithelial neoplasia grade 2 or 3 (CIN2/3), that can be treated and cured, to prevent progression to cancer. Most CIN2/3 lesions that are left untreated will progress to invasive cervical cancer. Current treatments for CIN2/3 in both high- and low-resource countries (LMICs) require trained health care providers, who are often out of reach for many women, particularly in rural areas in LMICs. Lack of access to precancer treatment following screening in LMICs in part accounts for the high burden of incident cervical cancer. Preclinical data have demonstrated pro-apoptotic effects of Artesunate (AS), a commonly available drug with an excellent safety profile in oral, rectal and intravenous routes primarily used to treat malaria in LMICS. This led to a recent Phase I study in the United States that demonstrated that self-administered vaginal artesunate inserts (pessaries) are safe, well-tolerated, and demonstrate efficacy for treatment of CIN2/3. Based on the mechanism of action, the clinical safety profile, and widespread availability as a generic drug on the World Health Organization (WHO) List of Essential Medications, vaginal artesunate inserts (pessaries), if backed by data from randomized trials, may offer patient-controlled and access cervical precancer treatment method for women in LMICs who face the greatest burden of cervical cancer and have difficulty accessing skilled providers for precancer treatment. However, given that artesunate is a well-known drug used in malaria treatment, it is critical to ensure that vaginal application of the drug will not promote resistance for use in malaria treatment.

**Objectives:** The proposed study seeks to investigate the pharmacokinetics of Artesunate (AS) and dihydroartemisinin (DHA), the active metabolite of artesunate, following intravaginal use at the dosing and frequency being studied for cervical precancer treatment. A secondary objective is to investigate safety among study participants.

**Methods:** As a recent study demonstrated safety and tolerability of intravaginal artesunate among women with CIN2/3 and the safety of artesunate is well documented in numerous malaria studies, the proposed study will be conducted in female volunteers regardless of their HPV or cervical precancer disease status. In this single arm study, blood levels of DHA and artesunate following 5 daily applications of vaginal artesunate inserts (pessaries) will be measured. A sample size of twelve participants (N=12) are needed. Up to 16 will be consented to account for drop offs. Eligible participants will self-administer artesunate vaginal inserts (pessaries) daily in the study clinic for 5 days. Participants will undergo a blood draw prior to receiving the first dose of artesunate, and on day 5 before inserting the vaginal insert, and at 15 min, 30 mins, 1, 2, 4, 6, and 8 hours after. In each patient, pharmacokinetic parameters of artesunate and DHA will be calculated via quantitative analysis of with determination of maximum concentration (Cmax), time to Cmax (Tmax), area under the serum concentration versus time curve (AUC), apparent clearance, and elimination half-life (t1/2).

**Budget:** The estimated study budget is US $70,000.

## **Protocol Summary**

| Protocol Title | Pharmacokinetics of intravaginal, self-administered Artesunate vaginal pessaries among women in Kenya. |
| --- | --- |
| Study Design | Single arm study of 12 participants who will use self-administered artesunate vaginal inserts (pessaries) on 5 consecutive days with blood draws performed at baseline and on day 5 for pharmacokinetic studies.  Up to 16 participants will be consented and enrolled, if necessary, to account for drop offs during the study protocol to ensure complete data for 12 participants. |
| Study Population | Women ages 18 or older meeting inclusion criteria. |
| Number of participants | A sample size of 12 is needed. Up to 16 participants will be consented, and enrolled, if necessary, to account for drop offs or incomplete data. |
| Number of sites | 2 |
| Phase of study (i.e., Pilot, Phase 1, etc.) | Phase I |
| Clinical Samples | Blood draw at Day 1 (Visit 1), and on Day 5 (Visit 5) at 0, 15 mins, 30 min, 1h, 2h, 4h, 6h and 8 h after self-administration of 200 mg intravaginal artesunate pessary. |
| Study Duration | 3 months |
| Participant  Duration | 2 weeks |
| Regimen or Intervention | 200 mg Artesunate vaginal insert (pessary) used daily for 5 consecutive days |

## **Lay Summary**

Cervical cancer, a disease of the cervix – the entrance of the womb is the second common cause of cancer in women around the world. Women in low- and middle-income countries like Kenya face the highest burden of cervical cancer. Cervical cancer is caused by human papillomavirus (HPV) infection. It can be prevented through vaccination against the HPV virus, and screening to find early changes in the cervix, called precancerous changes – scientifically called cervical intraepithelial neoplasia, which can be treated by a medical provider to prevent it from progressing to cancer. The HPV vaccines are new in Kenya and other low-income countries, and most women over 20 years of age have never been vaccinated. Screening for cervical cancer and treating precancerous lesions will prevent millions of unvaccinated women from getting diagnosed with cervical cancer. Current treatments for cervical precancer rely on a healthcare provider to perform them. In countries like Kenya and other LMICs where there are few nurses and doctors, many women found with precancer are often referred to far facilities to access treatment. Due to costs and other challenges, many women are unable to access referral centers with providers who can treat cervical precancer. Scientists are studying whether women with cervical precancer can use self-administered treatments with medications like a cream or pessary, to treat precancer, instead of relying on a health care provider to perform the treatment. One self-administered treatment being studied is a pessary formulation of the medication Artesunate, which is used to treat malaria in Africa and other places. A study in the United States has shown that Artesunate pessaries (vaginal inserts) are safe to use for precancer treatment, and ~ 70% women who used them were cured. Before Artesunate can be considered as a precancer treatment in Kenya and places like it, scientists need to understand whether using Artesunate as an intravaginal pessary can affect how it works to treat malaria – whether using it intravaginally can reduce its effect as a malaria treatment (developing resistance). This study is being done to investigate the blood levels of Artesunate when women use artesunate pessaries in the dosing being studied for cervical precancer. In other words, scientists are asking whether if a woman uses 200mg of artesunate pessary daily for five days, the medication can be found in the blood which could result in the medication being resistant to treating malaria. This is unlikely as the dosage used in the intravaginal pessary is much lower than the dosage used to treat malaria, and absorption of drugs in the vagina is much lower than when taken by mouth or used rectal – which is another way malaria drugs are given to children. To help answer this question of whether using low dose Artesunate pessaries for treating cervical precancer can cause resistance for malaria treatment, up to 16 female participants will be enrolled in this study. Study participants will use artesunate pessaries once daily for 5 days in the study clinic, and have blood drawn once on day 1 (prior to first pessary use), and six times on day 5 (last day of pessary use) to measure the artesunate drug levels in the blood. On day 5, participants will have a cannula placed and blood drawn from the cannula. The volume of blood drawn each time will be approximately 3 ml, less than a teaspoon. In comparison, when a person donates blood, approximately 500 ml of blood is drawn. The goal of this study is to confirm that artesunate pessaries can be used intravaginally for treatment of cervical precancer without creating resistance of the drug for treating malaria to enable studies on its use for precancer treatment.

# **1.0 LITERATURE REVIEW**

## **1.1 Intravaginal Artesunate for Cervical Precancer Treatment**

A potential topical therapy for which early studies are available is Artesunate (AS), a WHO-approved drug widely used to treat malaria in LMICs.^7^ Artesunate is a derivative of artemisinin the active component of Artemisia annua a plant used in Chinese traditional medicine for centuries. It is a well-known drug used in malaria treatment and its safety and tolerability has long been well established.^8–10^ Growing evidence has demonstrated that Artesunate has cytotoxic effects against numerous cancer cell lines both *in vitro* and *in vivo*.^11,12^ Proposed mechanisms of action include suppressing cell proliferation by inducing G1 and G2/M phase cell cycle arrest in the human breast, nasopharyngeal and renal cell cancer, as well as modulating key inflammatory pathways characteristic of uncontrolled proliferation and carcinogenesis.^13,14^ Ferroptosis – a type of iron-dependent cell death – is thought to be a key anticancer mechanism relevant to HPV-infected cells.^15^ Cancer cells are highly proliferative, requiring a heavy iron load which acts as a cofactor in synthesizing deoxyriboses before cell division.^16^ Development of both high-grade cervical intraepithelial neoplasia (CIN2/3), the precursor lesion of cervical cancer, and cervical cancer are associated with the expression of two viral proteins in the HPV lifecycle, E6 and E7.^17^ Epithelial cells that express either or both of these oncoproteins also overexpress the transferrin receptor, and have been shown to have increased levels of intracellular iron compared with normal cells.^15^

This observation has been exploited to investigate whether preinvasive cervical cancer (CIN2/3), can be treated with Artesunate, which contains an endoperoxide bridge that reacts with intracellular ferrous iron to generate free radicals, capable of inducing direct oxidative damage resulting in cell death.^14^ Given the mechanism of action, artesunate may provide beneficial and therapeutic effects for intraepithelial HPV disease. Given the unmet need for non-surgical treatment for cervical intraepithelial neoplasia (cervical precancer), and Artesunate’s demonstrated favorable safety profile for decades in both children and adults with malaria, a recent study (Trimble et al (2020),^18^ discussed below) has investigated its use as a nonsurgical, self-administered intravaginal treatment (as a vaginal insert/pessary) for HPV-associated disease.

### **1.1.1 Preclinical Studies**

#### **1.1.1.1 Mechanism of action**

Several studies demonstrate the pro-apoptotic effects of Artesunate by activating the mitochondria-dependent pathway, specifically activating caspase-3/9 and the release of cytochrome c from the mitochondria.^19^ Artesunate can also induce autophagy in uterine corpus endometrial carcinoma cells and by upregulating reactive oxygen species in human bladder cancer cells and hepatocellular carcinoma cells.^20,21,22^ Additionally, Artesunate has demonstrated anti-angiogenic properties through downregulation of VEGF and angiopoietin-1 in myeloma cells.^23–25^ Further, Artesunate is reported to have demonstrated anti-cancer effects such as formation of alkaline radicals via an endoperoxide bridge that reacts with intracellular ferrous iron leading to cell death.^26^

Because cervical squamous cell cancers and their precursors, cervical intraepithelial neoplasia (CIN), overexpress the transferrin receptor,^27^ these observations prompted a study of the cytotoxic effect of dihydroartemisinin (DHA), the bioactive form of Artesunate, on papillomavirus-expressing epithelial cells.^28^ *In vitro* studies demonstrated that, while DHA had little effect on normal cervical epithelial cells, it had a significant cytotoxic effect on HPV-immortalized cervical cells.^28^ Formulated as a local treatment (2.22 mg DHA dissolved in 100 µl dimethyl sulfoxide) in an oral mucosal canine nonclinical model which has 100% known tumor growth rate with HPV-infection, DHA has been reported to inhibit papillomavirus-induced tumor formation. In addition, tumor-negative dogs developed antibodies against the HPV L1 capsid protein.^28^

### **1.1.2 Clinical Studies**

#### **1.1.2.1 Safety and Toxicity for Intravaginal Use**

Intravaginal artesunate inserts (pessaries) has been tested in a multi-center dose-escalation phase I study to assess the safety, tolerability, and efficacy of vaginal artesunate in women with CIN2/3 in the United States (Trimble et al,(2020).^18^ In this ‘first-in-human’ study of intravaginal Artesunate for CIN2/3 treatment, treatment was safe, well tolerated, and reported adverse events (AEs) were all grade I or II and self-limited. Reported AEs among participants who used 3 five-day artesunate cycles included chills and flu-like symptoms (n=3, grade 1), vaginal (yeast) infection (n=1, grade II), dizziness or headache (n=2, grade 1), non-infective cystitis (n=1, grade 2), vaginal pain or uterine cramping (n=9, grade I), vaginal discharge (n=4, grade 1), vaginal pruritis (n=9, grade 1). In summary, 37 drug-related AEs were observed in this Phase I trial, of which 34 (92%) were grade I, and 3 (8%) were grade 2. Reported grade 2 adverse events included vaginal yeast infection (n = 6), bacterial vaginosis (n = 2), vaginal inflammation (n = 2), urinary tract infection (n = 2), and noninfective cystitis (n = 1). Events determined to be unrelated to the study medication included: anxiety, insomnia, suicidal ideation, vaginal twitching, fever, flu-like symptoms in a patient who developed a cold, body itching, chills, and eczema flare. No subjects withdrew from the study because of intolerable side effects, and all 28 subjects included in the modified-intention-to-treat analyses were able to complete their designated dosing regimen. No grade 3 or 4 adverse events were reported. A total of 3 subjects reported no noticeable side effects at all. Thus, the study drug to be used in this protocol (self-administered vaginal artesunate inserts) were shown to be safe and well-tolerated. A randomized, placebo-controlled phase II study of artesunate vaginal inserts for treatment of cervical intraepithelial neoplasia grade 2/3 (CIN2/3) is currently enrolling subjects at several US sites (NCT04098744). Patients enrolled to date reported no serious adverse events. A Phase II randomized trial of artesunate suppositories for the treatment of anal high-grade squamous intraepithelial lesions among HIV-negative individuals is similarly ongoing in the United States (NCT5555862)

## **1.2 Summary of Artesunate Pharmacokinetics**

**T**he pharmacokinetics (PK) measures of artesunate (AS) and dihydroartemisinin (DHA) following intravenous (IV), intramuscular (IM), oral and rectal administration have been well described.^29^ These PK parameters include maximum concentration of the drug in the blood (Cmax), time to maximum concentration Cmax (Tmax), apparent clearance (CL/F) - the drug concentration in the body in proportion to the rate of elimination, volume of distribution (V/F), the area under the serum concentration versus time curve (AUC) which expresses the total amount of the drug in systemic circulation after administration, and half-life (t_1/2_). Intravenous administration of AS produces quickly produces high maximum concentrations (Cmax) of AS, higher than any other method of administration. One example of this can be seen in a study of adults with uncomplicated malaria which compared AS and DHA levels following IV and IM administration, Cmax values for AS when administered intravenously reached over 16,000ng/mL while only reaching around 884ng/mL when administered intramuscularly.^30^ Similarly, the maximum concentration (Cmax) of AS and DHA peak quickly following IV administration, followed by a rapid decline. The average half-life of AS following IV administration of less than fifteen minutes in multiple studies, with an observed clearance range of 2-3 L/kg/hr and a volume range of 0.1-0.3 L/kg.^29^ The hydrolysis of AS into DHA following IV administration is similarly quick, with maximum DHA levels reached soon after IV AS administration. The Tmax for DHA after IV administration was consistently less than 25 minutes according to the observed studies with DHA clearance averaging between 0.5-1.5 L/kg/hr and volume averaging between 0.5-1.0 L/kg.^29^ The AUC of AS following IV administration of 120 mg AS in adults ranged between 876ng*hr/ml in healthy volunteers^31^ and 1038 - 1269ng*hr/ml in those with malaria.^32^ The AUC of DHA following IV administration of 120 mg AS in adults ranged from 1850 ng*hr/ml in healthy volunteers^33^ to 1845-2377ng*hr/ml in those with malaria.^34^

Compared to IV administration, IM administration produces lower peaks, longer half-life, and higher volumes of distribution for AS, as well as delayed peaks for DHA. For example, AS half-life following in IV administration is less than 15 minutes on average, compared to 25.2 to 48.2 minutes with IM administration.^29^ Other parameters – in including DHA half-life, volume of distribution, and clearance rates following IM administration resembled the values recorded after IV administration in multiple studies, due to the high bioavailability, assessed by exposure to DHA, associated with IM AS administration (>86%).^29^ The AUC of AS following IM administration of 120 mg AS in adults ranged between 856^35^ - 999^31^ng*hr/ml in those with malaria. The AUC of DHA following IM administration of 120 mg of AS in adults with malaria was 2474ng*hr/ml in one study.^31^

When AS is administered orally, DHA peak concentrations (Cmax), AUC, and half-life averages are all notably higher than comparable AS parameters. The average time to DHA Cmax and half-life following oral AS administration (200 mg/day) are 2 hours, and 0.5-1.5 hours, respectively, compared to one hour and 20-45 minutes, respectively, for AS.^29^ While a similar pattern is seen following IV and IM administration, namely, elevated levels of DHA half-life and AUC compared to AS, the difference are notable following oral administration. Also, while oral administration of AS results in a higher DHA Cmax compared to AS, IV and IM administration result in a notably higher AS Cmax than DHA Cmax. Morris et al (2011) points out that the variations observed following oral administration are most likely attributed to AS functioning as a “pro-drug” for DHA when ingested orally and in response to “first-pass or systemic metabolism.”^29^ Essentially, when AS is taken orally, it is converted to DHA at a greater extent than when it is taken intravenously or intramuscularly. The AUC of AS and DHA in a study of healthy adult volunteers taking 200mg AS daily for 5 days was 67 ng*hr/ml and 1158 ng*hr/ml for AS & DHA, respectively, on Day 1, and 60 ng*hr/ml and 1300 ng*hr/ml for AS & DHA, respectively, on Day 5.^36^ Similar Cmax parameters for AS were 67 ng/ml and 58 ng/ml on Day 1 and 5, respectively, and a pooled DHA Cmax of 654 ng/ml, demonstrating the absence of time-dependent artesunate pharmacokinetics in healthy subjects during 5-day oral administration of 200 mg artesunate.^36^ The AUC of AS following oral administration of 200 mg AS in adults ranged between 60-67ng*hr/ml in healthy volunteers^37^ and 310ng*hr/ml in those with malaria.^38^ The AUC of DHA following oral administration of 200 mg of AS in adults ranged from 1158-1331 ng*hr/ml ^37,39^in healthy volunteers to 3027ng*hr/ml in those with malaria.^38^ Rectal AS administration yields pharmacokinetic results similar to those obtained from oral administration, with the exception of delayed AS Cmax and longer AS half-life. Compared to IV administration, expectedly, both AS absorption and elimination are prolonged following rectal administration. Following rectal administration of AS, Tmax average between 0.58-1.43 hours, with a half-life between 0.9-.95 hours.^29^ These averages are based on three different studies: two studies containing pediatric patients with uncomplicated falciparum malaria (10 - 20mg/Kg dosing)^40,41^ and one study containing healthy Malaysian adults (200 mg rectal suppository, ~4mg/Kg dosing).^42^ Following rectal dosing of a one-time 200 mg AS suppository in healthy adults (similar to our planned dosing), a Cmax, Tmax, half-life and AUC of 448.5 ng/ml, 1.43 hours, 0.95 hours, and 796 ng*hr/ml of AS were observed, and Cmax, Tmax, half-life and AUC of 385.6 ng/ml, 1.80 hours, 1.21 hours, and 965 ng*hr/ml respectively of DHA were observed in healthy adults.^42^ No data are available on rectal PK in adults with malaria as this route of administration is not used to treat malaria in adults. The longer half-life of AS following rectal dosing (average 0.9-0.95 hrs) compared to IM (average 25.2 – 48.2 minutes), or IV (average less than 5 minutes) may reflect absorption rate-limited elimination of AS.^29^ As is expected given that rectal AS administration avoids by-pass metabolism, the discrepancy in AS and DHA AUC values (796 ng*hr/ml and 965 ng*hr/ml, respectively), is not as striking with rectal, as compared with oral administration of AS (119 ng*hr/ml and 1331 ng*hr/ml, respectively). ^29^ Similar to oral administration, both DHA Tmax and half-life values were higher than that of AS following rectal administration.^29^

## **1.3 Effect of malaria infection status on artesunate and DHA pharmacokinetics**

A direct comparison among healthy and parasitaemic subjects was conducted by Teja-Isavadharm et al, who studied the PK of DHA following oral AS administration in six healthy adults and six adult falciparum malaria patients.^43^ The investigators determined that AUC and Cmax of DHA were significantly higher in subjects with malaria as compared to healthy subjects. Binh et al obtained similar results when comparing the PK in eight patients with falciparum malaria and ten healthy subjects.^44^ However, given the relatively small size of both studies, definitive conclusions regarding differences in PK between healthy and infected subjects cannot be drawn at this time.^33^ However, as DHA clearance is dependent on hepatic blood flow, a reduction in clearance, and consequently an increase in exposure associated with acute malaria infection would be consistent with known DHA’s PK properties.^33^

## **1.4 Malaria treatment and artemisinin partial resistance**

The World Health Organization recommends artemisinin- based combination therapies (ACTs) as the first line treatment for uncomplicated Plasmodium Falciparum.^45^ In ACTs, artemisinin quickly and drastically reduces the majority of malaria parasites, while the partner drug clears the remaining parasites to prevent recrudescence.^46,47^ Artemisinin partial resistance is defined as delayed parasite clearance following treatment with an artesunate monotherapy or ACT, and can be observed as a parasite clearance half-life of greater than five hours, or parasites microscopically evident on day three.^48^ This represents partial resistance. Artemisinin partial resistance alone does not necessarily lead to malaria treatment failure. However, reduced efficacy of the artemisinin component places greater demands on the partner drug to clear a larger parasite mass, jeopardizing future efficacy of the partner drug (WHO). Examples of ACTs include artemether-lumefantrine, artesunate-amodiaquine, artesunate-mefloquine, among others. Standard ACTs regimen for uncomplicated malaria is an oral 3-day course. Most studies show that ACTs currently recommended in national malaria treatment policies remain effective, with an overall efficacy rate of greater than 95%.^49^

Artemisinin’s are exceptionally fast acting against intra-erythrocytic asexual blood-stage malaria parasites, effecting up to 10,000- fold reductions in parasite burden every 48 hours.^50^ The primary genetic drivers of artemisinin partial resistance, both *in vitro* and *in vivo* are point mutations in in the P. falciparum Kelch13 (*PfK13*) gene in the early ring stage.^51,52^ K13 mutations allow a subset of early ring-stage parasites to survive cell-cycle arrest brought on by artemisinin exposure, enabling those parasites to reinitiate transcription and complete their intraerythrocytic developmental cycle once artemisinin is no longer present at inhibitory concentrations.^53^ Partial resistance *in vitro* is routinely defined as greater than 1% survival of early ring-stage parasites exposed for 6 hours to 700nM dihydroartemisinin (DHA-the primary active metabolite of ART) followed by drug-free culture incubation for a further 66 hours.^48^ The mechanism of partial resistance appears to involve a complex interplay of K13 protein abundance, hemoglobin endocytosis, and the parasite response to stress.^48,54^

In Africa, several studies have identified a number of low frequency Pfk13 mutations.^55,56^ The mutations known to be associated with delayed parasite clearance (M476I,P553L,R561H, P574L, C580Y and A675V) were observed at low frequencies (<5%) in four countries, Ghana, Rwanda, Uganda and Tanzania.^55^ In Tanzania, two parasites sampled from 764 (0.3%) were identified in 2017, [^11^](https://sciwheel.com/work/citation?ids=10857934&pre&suf&sa=0) and a single parasite from 422 samples in 2019 (0.2%).^55^ Similarly in Uganda one parasite was identified from 796 samples in 2018/2019 (0.1%).^57^ In a 2021 study evaluating artemisinin partial resistance in Northern Uganda conducted between 2017 to 2019, a total of 14 of 240 patients who received intravenous artesunate had evidence of in vivo artemisinin partial resistance (parasite clearance half-life >5 hours).^57^ Of these 14 patients, 13 were infected with P. falciparum parasites with mutations in the A675V or C469Y allele in the kelch13 gene.^52^

P. falciparum partial resistance to artemisinin has been documented in five countries in Southeast Asia; Cambodia, Lao People’s Democratic Republic, Myanmar, Thailand and Vietnam (WHO).^47^ With implementation of combination therapy, improvements to health systems and surveillance systems to monitor first- and second-line treatment, the consequences of the development of partial resistance to antimalaria medicines may be less severe today than what was observed with chloroquine in the 1980s. In the event parasites develop reduced sensitivity to artemisinin, ACTS will continue to cure malaria, provided that the partner drug remains effective.^47^ Potential changes to ACT to overcome resistance include modifications such as extending the duration of the ACT course (currently 3 days for oral treatment), alternating use of different ACT regimens, and addition of another antimalarial drug to the standard ACTs (triple-ACT).^47^ Further, a malaria vaccine (e.g. RTS, S vaccine) could be added to mass drug administration campaigns to enhance the treatment efficacy and to prevent further artemisinin partial resistance development.

## **1.5 Systemic Artesunate absorption and possible implications for developing resistance for malaria treatment.**

While there are no pharmacologic data on serum absorption following intravaginal artesunate administration – hence this study, direct application of artesunate to the cervical mucosa at the proposed dose is unlikely to result in systemic absorption. The planned intravaginal artesunate dose of 200 mg (≤4 mg/kg based on planned inclusion criteria of weight≥ 50 Kg) is 2.5 fold lower than the approved rectal suppository dose (10mg/Kg). Unlike rectal mucosa which is highly vascular and comprised of a single-cell layer of columnar epithelium making it highly permeable to drugs, resulting in systemic absorption and hence use of rectal artesunate for pre-referral pediatric malaria treatment, the cervico-vaginal tissue is comprised of a thick, stratified squamous epithelial cell layer,58 and is significantly less vascular, reducing systemic absorption. Similarly, due to the rapid rate of elimination Artesunate’s active metabolite,59 no systemic accumulation is expected of Artesunate or its active metabolite with intravaginal multi-day dosing in the context of cervicovaginal administration.

# **2.0 BACKGROUND AND RATIONALE**

## **2.1 Background**

Although cervical cancer, defined as cancer occurring in cells of the cervix is preventable, low- and middle-income countries (LMICs) bear a disproportionate burden, accounting for 85% of an estimated 570,000 incident cases, and 90% of deaths annually.^1^ In Kenya, cervical cancer accounts for 5,240 (12.9%) of new cancer cases annually and 3,286 (11.4%) of all cancer deaths annually.^2^ Cervical cancer is caused by infection with human papillomavirus (HPV), and primary prevention is achieved through vaccination against the HPV virus. Secondary prevention of cervical cancer is achieved by regular screening during which time the cervical precancer lesion – cervical intraepithelial neoplasia – can be detected and treated to prevent progression to cervical cancer. Both cervical precancer and cancer diagnoses are established on pathology evaluation of cells from the cervix obtained through a biopsy. The standard of care in Kenya and other LMICs for treatment of cervical precancer, recommended by the WHO, is ablation using cryotherapy or thermal ablation, or excision – using the loop electrosurgical excision procedure (LEEP) or cold knife cone.^7^ Current cervical precancer treatment methods rely on access to a trained healthcare provider and access to ablative or excisional equipment, both of which can be challenging to access in countries with high patient to provider ratios and weaker health systems. This causes delays in treatment access in settings where women have to travel far to reach a trained healthcare worker, leading to missed opportunities for cancer prevention, resulting to progression to cancer. In a study from western Kenya, when women who screened positive for cervical precancer in peripheral clinics were referred to a central/referral clinic for treatment, 39% were not able to make their referral appointment. Additionally, excisional treatments are associated with obstetric consequences including preterm birth. To mitigate challenges related to access to provider-administered cervical precancer treatment in low-resource countries, scientists are investigating use of non-excisional treatments including topical, self-administered therapies with cytotoxic properties for treatment of cervical precancer. Recent evidence, primary from studies in high-income countries (HICs), demonstrate that topical therapies – which can both be self- or provider-administered - may be utilized as treatment of HPV-associated anogenital lesions including cervical precancer.^2–6^

# **2.2 Problem Statement and Study Rationale (Justification)**

Given that artesunate is a well-known drug used in malaria treatment, it is critical to ensure that vaginal application of the drug for treatment of cervical precancer will not promote malaria resistance. Thus, a study evaluating blood serum levels after artesunate vaginal inserts administration is proposed. In this study, participants will self-administer one 200 mg artesunate vaginal insert per day for 5 days. Blood serum levels of DHA and artesunate will be evaluated prior to receiving artesunate, and on day 5, before artesunate vaginal insert (pessary) use – time 0, and at 15min, 30min, 1, 2, 4, 6 and 8 hours after use to evaluate the pharmacokinetics of artesunate following intravaginal use at the proposed dose and frequency.

# **3.0 RESEARCH HYPOTHESIS AND OBJECTIVES**

## **3.1 Research Hypothesis**

The overall objective is to evaluate pharmacokinetics (PK) of intravaginal, self-administered Artesunate vaginal inserts (pessaries) among women in Kenya by measuring serum DHA and artesunate levels after intravaginal administration to evaluate for potential risk of developing malaria resistance with artesunate use for cervical precancer treatment.

The central (null) hypothesis is that there will be no significant difference in detectable levels of blood DHA and artesunate following 5 consecutive days of intravaginal, self-administration of 200 mg artesunate pessaries, compared to baseline

## **3.2 Research Objectives**

**Primary Objective:** To determine the area under the serum concentration versus time curve (AUC) of dihydroartemisinin (DHA) following five consecutive days of self-administration of 200mg Artesunate vaginal inserts (pessaries) among healthy women in Kenya.

**Secondary Objective**

1. To determine the area under the serum concentration versus time curve (AUC) of Artesunate (AS) following five consecutive days of self-administration of 200mg Artesunate vaginal inserts (pessaries) among healthy women in Kenya
2. To determine the maximum concentration of Artesunate (AS) (Cmax) following five consecutive days of self-administration of 200mg Artesunate vaginal inserts (pessaries) among healthy women in Kenya
3. To determine the maximum concentration of dihydroartemisinin (DHA) (Cmax) following five consecutive days of self-administration of 200mg Artesunate vaginal inserts (pessaries) among healthy women in Kenya
4. To determine the time to maximum concentration (Tmax) of Artesunate (AS) following five consecutive days of self-administration of 200mg Artesunate vaginal inserts (pessaries) among healthy women in Kenya
5. To determine the time to maximum concentration (Tmax) of dihydroartemisinin (DHA) following five consecutive days of self-administration of 200mg Artesunate vaginal inserts (pessaries) among healthy women in Kenya
6. To determine the half-life (t_1/2_) of Artesunate (AS) following five consecutive days of self-administration of 200mg Artesunate vaginal inserts (pessaries) among healthy women in Kenya
7. To determine the half-life (t_1/2_) of dihydroartemisinin (DHA) following five consecutive days of self-administration of 200mg Artesunate vaginal inserts (pessaries) among healthy women in Kenya
8. To determine the apparent clearance (CL/F) of Artesunate (AS) following five consecutive days of self-administration of 200mg Artesunate vaginal inserts (pessaries) among healthy women in Kenya
9. To determine the apparent clearance (CL/F) of dihydroartemisinin (DHA) following five consecutive days of self-administration of 200mg Artesunate vaginal inserts (pessaries) among healthy women in Kenya
10. To determine the volume of distribution (V/F) of Artesunate (AS) following five consecutive days of self-administration of 200mg Artesunate vaginal inserts (pessaries) among healthy women in Kenya
11. To determine the volume of distribution (V/F) of dihydroartemisinin (DHA) following five consecutive days of self-administration of 200mg Artesunate vaginal inserts (pessaries) among healthy women in Kenya
12. To investigate the safety of 5-day course of self-administered intravaginal artesunate vaginal inserts (pessary) in women in Kenya

## **3.3 Conceptual Framework**

Conceptual framework of artesunate action on HPV-infected cells, specifically targeting E6 & E7 oncogenes/oncoproteins, which forms the basis of current clinical trials studying use of this compound for cervical precancer treatment. Image adapted from Bedell et al (2020)


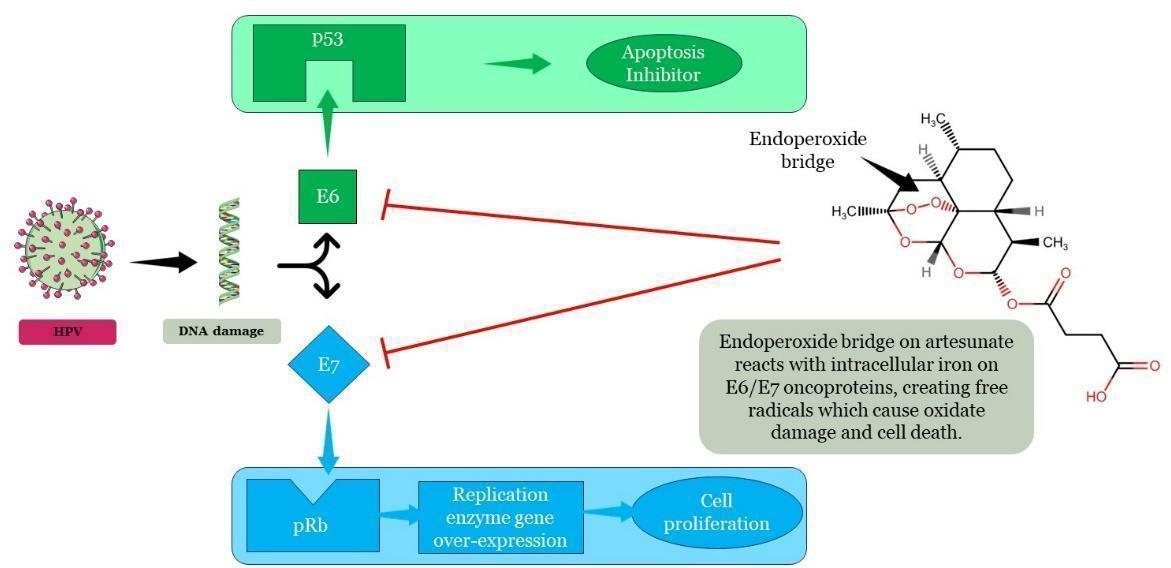


## **3.4 Study Schema**

**Screening/Enrollment Visit**

Review eligibility criteria, review and sign informed consent, introduce and discuss study requirements, urine pregnancy test, malaria antigen testing, assign unique study number, obtain baseline clinical and demographic characteristics and medical history, pelvic examination.

↓

**Visit 1/Day 1* (may be combined with Screening/Enrollment visit)**

Interval medical history, urine pregnancy test

***Blood draw*** for baseline Artesunate/DHA testing

Self-administration of first Artesunate vaginal insert (pessary) and tampon, followed by 30-minute observation.

Schedule Visit 2

↓

**Visit 2-4/Day 2-4**

Interval medical history, urine pregnancy test, review adverse events

Self-administration of Artesunate vaginal insert (pessary) and tampon

Schedule subsequent visit

↓

**Visit 5/Day 5**

Interval medical history, urine pregnancy test, review adverse events

***Blood draw*** before artesunate administration (time 0) Self-administration of Artesunate vaginal insert (pessary)

***Blood draw*** at 15 min, 30 min, 1hr, 2hr, 4 hr, 6 hr and 8 hrs after Artesunate vaginal pessary insertion

Schedule Visit 6

↓

**Visit 6 (within 4-10 days of Visit 5)**

Review adverse events

Study exit

## **3.5 Rationale for Study Design**

The study is designed to evaluate pharmacokinetic of intravaginal, self-administered Artesunate, with the hypothesis that there will be no significant difference in detectable levels of serum DHA and artesunate following intravaginal use at the proposed dosage and frequency compared to before administration. The timing of the serum blood draws and hence PK analysis parameters were chosen based on the dosing regimen of intravaginal artesunate for cervical precancer treatment used in ongoing clinical trials. Current studied regimens use a 200 mg vaginal insert/pessary, used daily for 5 applications, followed by one week off, repeated for 3-4 cycles.

The rationale for PK analysis – obtaining a pre-administration artesunate level before day one, followed serial blood draws on day 5 only one cycle of intravaginal artesunate use is based on available pharmacologic data regarding half-life of artesunate and DHA,^29^ and expected lack of accumulation following a one-week wash off period, in case of multiple artesunate cycles administered as is currently being studied. While there are no published pharmacological data for intra-vaginal administration of artesunate suppositories, clinical pharmacology of artesunate and its metabolite DHA following rectal, IV, IM, and oral administration are well documented in the malaria literature.^60,61,62,29^ Artesunate is rapidly absorbed, with peak serum levels occurring 1.5h, 2h, and 0.5h after oral,^63^ rectal,^30,64^ and intramuscular^30^ absorption, respectively. Artesunate is almost entirely converted to its active metabolite DHA.^65^ Elimination of artesunate is very rapid, and its activity is determined by DHA elimination, which has a half-life of 0.5-1.5 hours.^59,29^

In prior studies investigating multiple daily artesunate IV use in healthy subjects, Artesunate drug concentrations did not demonstrate accumulation, consistent with known rapid elimination rates of artemisinin derivates.^62^ Following IV injection, artesunate drug levels rapidly declined and was converted to dihydroartemisinin (DHA) with overall mean elimination half-lives ranging from 0.15 – 0.23 hr for artesunate and 1.23 – 1.63 hours for DHA.^29^ The differences between Artesunate and its principal metabolite are due to a combination of differences in solubility and to rapid conversion of Artesunate to DHA.^30^ Despite the prediction of low systemic concentration of the drug, rapid elimination following intravaginal administration would be expected, as seen with IM administration.^30^ Similarly, a significantly lower maximum serum concentration after intra-vaginal administration is expected, compared to IV administration, or rectal administration given higher vascularity of rectal mucosa compared to cervicovaginal mucosa. Unlike vaginal tissue which consists of a thick, stratified squamous epithelial cell layer,^58^ rectal mucosa has only a single-cell layer of columnar epithelium which is 6-8 fold thinner than the vaginal epithelial layer, making it more permeable to drugs.^66^ The endocervical canal is lined by columnar epithelium, and will likely be exposed to the drug and hence impact absorption. However, we anticipate the concentrations of the drug exposed to the endocervical canal of a closed cervix to be much lower than that exposed to the vaginal tissue and ectocervical canal which will be directly exposed to the study drug.

Current precancer studies dose intravaginal artesunate for 5-days on Week 1, 3, and 5, or Week 1, 3, 5, 7, with Weeks 2, 4, 6, or Weeks 2, 4, 6, 8 drug-free, respectively. That is, each 5-day cycle is followed by a one week (at least 7 days) of no drug use in between each cycle. Given the short half-life of DHA following daily dosing of Artesunate in adults, we expect no accumulation after each 5-day cycle when following a drug-free week. As such, this study will measure blood serum levels of DHA and artesunate following only one 5-day cycle of daily applications of 200 mg artesunate pessaries in studying PK of the artesunate pessaries following intravaginal use for this indication.

# **4.0 STUDY DRUG AND ADMINISTRATION**

## **4.1. Description of the Study Drug**

In this study, a vaginal insert (pessary) formulation of artesunate – made by Frantz Viral Therapeutics- will be used. In LMICs including Kenya, commercially available Artesunate suppository is approved for rectal use for pre-referral malaria treatment.^67^

**Description of Artesunate – the active ingredient**

Artesunate is a derivative of artemisinin which is the active principle of the Chinese medicinal herb *Artemisia annua*.

Nomenclature: Artesunate, Artesunic acid

Chemical Name: [3*R*,5a*S*,6*R*,8a*S*,9*R*,10*S*,12*R*,12a*R*)-3,6,9-trimethyldecahydro-3,12-epoxypyrano[4,3-*j*]-1,2-benzodiox epin-10-ol, hydrogen succinate;

CAS Reg. No.: 88495-63-0

General Properties

Molecular Formula C_19_H_28_O_8_

Molecular Weight 384.4 g/mol

Physical Description A fine, white crystalline powder

Solubility Very slightly in water; very soluble in dichloromethane; freely soluble in ethanol (~ 750 g/l) and acetone

Melting Point 132-135ºC

Optical Rotation R; [α] D 20 °C = +2.5° to +3.5° in a 10mg/mL in dichloromethane pH 3.5-4.5 in an aqueous suspension containing 10 mg/g

Artesunate vaginal inserts composition can be found in the table below

**Quantitative composition of artesunate insert**

| **Ingredient** | **Function** |
| --- | --- |
| Artesunate | Active ingredient |
| PCCA MBK^TM^* - hydrogenated vegetable oil and PEG-8 distearate. | Base |
| Butylated Hydroxyl Anisole NF | Preservative |

**Supplier/How Supplied, Formulation**

Artesunate vaginal inserts will be produced under controlled environmental conditions as prescribed in USP Chapter 795.

Clinical trial supplies will be produced by Buderer Drug Co. in Perrysburg, Ohio. Buderer maintains compliance with USP Chapter 795 (Pharmaceutical Compounding – Nonsterile Preparations) and USP Chapter 797 (Pharmaceutical Compounding – Sterile Preparations) which establish practice standards and outline the responsibility of the compounder, selection and appropriate sources of ingredients, quality control, and considerations regarding the stability of compounded preparations. Two additional USP informational chapters are in effect, including, USP Chapter 1075 – Good Compounding Practices, and USP Chapter 1160 – Pharmaceutical Calculations in Prescription Compounding.

## **4.2 Dosage and Administration**

Dose: 200 mg daily for five consecutive days

Route of administration: Intravaginal, using an intravaginal applicator

**Packaging and Labeling:** Pessaries (vaginal inserts) for dispensing will be packaged in standard prescription use plastic or cardboard containers. Proper labeling will be applied per state regulations.

**Storage and Stability:** The inserts may be stored at controlled room temperature. Inserts for dispensing will be packaged in a plastic or cardboard container and kept in their plastic mold until administration

**Administration Instructions:** All study participants will receive detailed counseling and instructions on artesunate use prior to administration, including instruction to wash their hands before and after inserting the pessaries. At the time of visit 1, after a negative pregnancy test is confirmed, the study physician or nurse will provide the first vaginal insert, applicator, and tampon. During that visit, the subjects will be instructed on the vaginal application of Artesunate inserts using the applicator provided, followed by the study-provided tampon. Tampons should be kept in place after each artesunate insert for at least 4 hours, but no longer than 10 hours. Tampons should be discarded properly, away from children and pets. Once the tampon is removed, participants will be advised to thoroughly wash their hands, and wipe outside of their genital area (vulvar and perianal) to remove any residue of the drug. To minimize irritation, participants will be advised not to engage in sexual contact (genital, anal, or oral sex) for at least 4 hours after applying the vaginal inserts, on days 1-5. Participants will return to the study clinic daily for visits 2-5 for self-administration of inserts numbers 2-5 as well as for review of any adverse events. Participants will be advised not to douche during the five days of intravaginal artesunate use, and not to use any intravaginal ointments, gels, or other types of pessaries. For any bacterial or yeast vaginal infections, participants should use oral medication, which will be prescribed by the study doctor.

**Dose rationale:** The dose for this study (200 mg) was chosen based on a combination of published clinical and pharmacokinetic data regarding intra-rectal administration of artesunate suppositories setting.^68^ along with clinical data demonstrating the safety, tolerability and efficacy of this dosing for treatment of cervical precancer (CIN2/3) in a U.S Phase I trial.^18^ This same dosing is being studied in ongoing Phase II trials.

**Comparison of study dose to artesunate dosage for other indications:** The proposed study dose of 200 mg per pessary (≤4 mg/Kg for participants who weight ≥50 Kg, which is an inclusion criteria for the study) is considerably less than doses associated with toxicity in animal studies (> 60 mg/Kg) and the rectal suppository doses used in children (approximately 10mg/Kg) and adults (approximately 8.3 mg/Kg) for malaria treatment.35 This provides additional rationale for safety.

## **4.3 Accountability Procedures for Study Investigational Drug**

**Storage, Stability and Handling:** Artesunate pessaries will be stored at room temperature at the site pharmacy with controlled access and dispensed under the authority of a licensed pharmacist. Pessaries/inserts for dispensing will be packaged in a plastic or cardboard container and kept in their plastic mold until administration. Any unused pessaries will be returned to the study site pharmacy for segregation and destruction as per the Pharmacy and Poisons Board guidelines on pharmaceutical waste management and disposal.

**Study Drug Accountability:** Following regulatory approval, drug supply will be shipped to the Institutional Pharmacy. The investigator or designee is responsible for keeping accurate records of the clinical supplies received from the company sponsor or designee, the amount dispensed to the subjects and the amount remaining at the conclusion of the trial. The number of study drug dispensed will be recorded on the Investigational Drug Accountability Record (Appendix A).

**Concomitant Medications:** During the 5-day dosing phase (Visit 1 – Visit 5), participants are prohibited from using artemisinin-based therapies including for malaria treatment. Participants diagnosed with malaria during this short time will be prescribed alternative evidence-based therapy. Use of efavirenz antiretroviral therapy is also prohibited during the dosing phase and is an exclusion criteria.

# **5.0 METHODOLOGY**

## **5.1 Study Details and Procedures**

**Study Design and Sampling**: This is a single-arm, open-label Phase I, non-randomized study. Participants meeting the inclusion criteria will be sequentially enrolled.

**Study Population:** The study population will be up to 16 women 18 years or older living in Kisumu County in Kenya, in order to have 12 fully evaluable participants, our desired sample size. Fully evaluable participants are those who receive artesunate inserts for 5 consecutive days and undergo blood draws before and after receiving artesunate at all the timepoints per study protocol. Enrollment will stop once data from 12 fully evaluable participants are obtained. No more than 16 participants will be enrolled.

**Inclusion Criteria**

- Age 18 years to 65 years
- Negative pregnancy test at screening
- Weight ≥50 Kg at study entry*
- Willingness to use contraception (hormonal or barrier) during the 5-day study dosing phase if of childbearing age (less than 50 years of age)
- Ability and willingness to provide informed consent
- Plan to reside in the study location during the study period
- Agrees for samples without identifiers to be shipped outside of Kenya for testing

*Justification for weight criteria: A minimum body weight of 50Kg will meet the planned artesunate dosing of ≤4 mg/Kg for which excellent safety data is available.

**Exclusion Criteria**

- Current pregnancy or breastfeeding status
- History of total hysterectomy
- Known allergy to Artesunate
- Have a medical comorbidity that in the opinion of the investigator would interfere with study participation
- Currently receiving artemisinin-based agents for malaria treatment or completed artemisinin-based treatment within the previous 3 days.*
- Male at birth
- Current use of efavirenz antiretroviral therapy, phenytoin, carbamazepine, and rifampin
- Positive malaria antigen test at screening

*Based on dihydroartesunate (artesunate’s active metabolite) half-life of between 0.5-1.5 hours^29^

**Study Location:** The study will be carried out at the Lumumba Sub-County Hospital and at the Victoria Biomedical Research Institute in Kisumu, Kenya. All study activities will take place at Lumumba Sub-County Hospital except day 5 study visit, which will take place at Victoria Biomedical Research Institute.

**Recruitment:** Participants will be recruited from among women in the general population within the vicinity of the study location, including within health facilities in Kisumu County in Kenya. The study team will include a brief description of the study as part of outreach activities and educational talks at surrounding facilities. Participants interested in participating will be screened for eligibility individually. The informed consent and all study procedures will be carried out in a private area before any study activities are performed.

**Schedule of Events**

|  | **SCREENING/ENROLLMENT VISIT** | **Visit 1^1^** | **Visits 2-4** | **Visit 5** | **VISIT 6^2^** |
| --- | --- | --- | --- | --- | --- |
| Informed Consent | **X** |  |  |  |  |
| Assess Eligibility | **X** |  |  |  |  |
| Review and record demographic and medical History | **X** |  |  |  |  |
| Assign unique participant ID number | **X** |  |  |  |  |
| Pelvic exam  Malaria antigen testing^2^  Urine pregnancy testing | **X** |  |  |  | **X** |
|  | **X** |  |  |  |  |
|  | **X** | **X** | **X** | **X** |  |
| Collect locator information | **X** | **X** | **X X** | **XX** |  |
| Demonstration of intravaginal *Artesunate* application using pelvic model | **X** | **X** | **X** | **X** |  |
| Artesunate pessary self-administration in clinic |  | **X** | **X X** | **XX** |  |
| Review concomitant/prohibited medications | **X** | **X** | **X** | **X** |  |
| Blood Draw(s) |  | **X** |  | **XX** |  |
| Adverse events review |  | **X** | **X X** | **XX** | **XX** |
| Transport Reimbursement | **X** | **X** | **X X** | **XX** | **XX** |
| Study exit |  |  |  |  | **XX** |

1.Visit 1 may be combined with the screening visit

2. Those positive will be referred for treatment and be screen failure

*Visit 6 will occur 4-10 days after visit 5

## **5.2 Study Activities by Visit**

**Pre-Screening:** As part of participant outreach and recruitment, study staff can pre-screen potential study participants at either on-site or off-site locations. During these interactions, study staff may explain the study to potential participants and ascertain elements of presumptive eligibility to be confirmed at an on-site screening visit. Process information (e.g., number of potential participants contacted, number presumptively eligible) may be recorded and stored at study sites in the absence of written informed consent from potential participants, provided the information is collected in such a manner that it cannot be linked to participant identifiers.

**Screening/Enrollment Visit:** The screening period may be up to 14 days prior to Visit 1. Multiple visits may be conducted to complete all required screening procedures, if necessary. Written informed consent for screening/enrollment must be obtained before any screening procedures are initiated. For participants who do not meet the eligibility criteria, screening will be discontinued once ineligibility is determined, and a screen failure recorded. As part of screening activities, participants will have a malaria antigen test to screen for subacute malaria infection. Those who test positive will be referred for treatment and considered screen failures. Given a malaria-endemic region, we expect and plan for a high screening to enrollment ratio because of the probability of high rates of sub-clinical malaria. Variables to be collected will include basic demographic (e.g., age, education level, marital status) and clinical (e.g., gravidity and parity, contraception use, pregnancy testing) variables as well as symptom review will be collected.

**Screening/enrollment visit activities:**

- Confirm eligibility
- Obtain informed consent
- Assign unique participant ID number
- Study protocol counselling (introduce the study to the participant, explain all study activities and timing, length, expectations of the participant, demonstration of intravaginal *Artesunate* application using pelvic model, discussion of tampon use etc.)
- Perform limited physical and pelvic exam
- Collect locator information
- Urine pregnancy testing
- Review concomitant/prohibited medications
- Obtain basic demographic and clinical data

**Visit 1**: Visit 1 can occur on the same day or up to 14 days following the screening/enrollment visit. Visit 1 will be timed to be no less than 1 week before the participant’s anticipated menstrual cycle, for menstruating participants.

**Visit 1 activities:**

- Confirm eligibility
- Update locator information (if necessary)
- Review artesunate self-administration instructions and study protocol
- Review concomitant/prohibited medications
- Draw blood (~2.5ml) before artesunate self-administration
- Artesunate self-administration using applicator, followed by tampon
- Observation for 30 minutes
- Provide transport reimbursement
- Schedule Visits 2

**Visit 2-4:** Visits 2, 3 and 4 must occur sequentially (within 24 hours) of each other. Attempts will be made to start visits at approximately the same time each day.

**Visit 2-4 activities:**

- Update locator information (if necessary)
- Review and record adverse events using standardized questionnaire
- Perform pelvic exam as needed based on AEs described (pelvic exam for suspected grade II or worse AEs)
- Review concomitant/prohibited medications
- Artesunate self-administration using applicator, followed by tampon insertion
- Observation for 30 minutes
- Provide transport reimbursement
- Schedule subsequent visit

**Visit 5:** Visit 5 will occur one day (~24 hours) after Visit 4. Attempts will be made to start visits at the same time each day.

**Visit 5 activities:**

- Update locator information (if necessary)
- Review and record adverse events using a standardized questionnaire
- Perform pelvic exam as needed based on AEs described (pelvic exam for suspected grade II or worse AEs)
- Review concomitant/prohibited medications
- Draw blood (~2.5ml) before artesunate self-administration & insert peripheral cannula for subsequent blood draws
- Artesunate self-administration using an applicator, followed by tampon insertion
- Blood draws (~2.5ml each time) at 15, 30min, 1h, 2h, 4h, 6h, and 8h after artesunate self-administration.
- Provide transport reimbursement
- Schedule visit 6

**Visit 6:** Visit 6 will occur 4-10 days after visit 5

**Visit 6 activities:**

- Review and record adverse events since last visit using standardized questionnaire
- Study exit/termination
- Provide reimbursement

## **5.3 Adverse Event Reporting**

**Definitions**

An adverse event (AE) is any untoward medical occurrence in a patient or clinical investigation participant administered a pharmaceutical product and which does not necessarily have a causal relationship with this treatment. An adverse event can therefore be any unfavorable and unintended sign (including an abnormal laboratory finding), symptom, or disease temporally associated with the use of a medicinal (investigational) product, whether or not related to the medicinal (investigational) product.

An unexpected (unlisted) adverse event is an adverse reaction, the nature or severity of which is not consistent with the applicable product information (e.g., Investigator’s Brochure for an unapproved investigational product or package insert/summary of product characteristics for an approved product).

A serious adverse event (SAE) is any untoward medical occurrence that at any dose:

- results in death;
- is life-threatening;
- requires inpatient hospitalization or prolongation of existing hospitalization;
- results in persistent of significant disability/incapacitation; or
- is a congenital anomaly/birth defect

Important medical events as assessed by medical and scientific judgment may also be considered SAEs by the investigator and should be reported in an expedited fashion.

**Safety/Adverse Events Evaluations:** Safety evaluation to evaluate for adverse events will be done using U.S National Cancer Institute Common Terminology Criteria for Adverse Events, v5.0 (CTCAE 5.0) (Appendix B) for systemic AEs^69^ and the Division of AIDS Female Genital Grading Table for Use in Microbicide Studies (Appendix C) for localized/genital AEs.^70^Adverse events will be elicited by a trained clinician (nurse, clinical officer, or medical officer) at the study visits 1 through 6. As part of AE evaluation, systemic symptoms that may be related to study drug use including headache, dizziness, tinnitus, GI discomfort will be evaluated. Localized/pelvic AEs including vaginal pruritis, discharge, pelvic pain will also be elicited during study visits. All participants will have a pelvic and speculum exam at baseline to document the appearance of genital mucosa before artesunate administration. During visits 1-5, pelvic/speculum exam will be performed based on clinical indications only, including among all participants reporting suspected grade 2 or higher local AEs including pain, irritation, itching, or erythema, based on the Division of AIDS Female Genital Grading Table for Use in Microbicide Studies standardized grading scale. Clinicians will have a low threshold for performing a pelvic exam during these visits based on participant symptoms. At visit 6 (study exit), all participants will have a pelvic exam to document appearance of genital mucosa at end of artesunate use. During pelvic examinations, the vulva, vaginal tissue and cervix will be inspected for evidence of inflammation or erythema. Per the CTCAE and Division of AIDS standardized grading scales, AEs will be graded as grade 0- none, grade 1 – mild, grade 2 – moderate, grade 3 – severe, grade 4- potentially life-threatening, or grade 5 – death-related AEs.

**Management and reporting of adverse events:** Based on the prior Phase I study of intravaginal artesunate at this dosage and frequency, self-limited grade 1 (mild) AEs are expected, and grade II (moderate) AEs will be few, while grade 3 or worse AEs are not expected. In this study, AEs will be elicited and recorded in the participant study records at each study visit. All local AEs will be considered causally related to administration of the study drug. Causality of all other AEs will be assessed individually. Participants with grade I or II AEs will be followed until resolution of AEs. Grade II AEs will be managed symptomatically. Vaginal infections including yeast infections will be treated with oral medications during the dosing phase. Significant local inflammation, if any, will be treated with oral analgesics. Local infection or ulceration, which are unexpected, will be treated with routine wound care and antibiotics as necessary. Participants found to have any grade 3 or higher AEs will be instructed to stop study drug use, and the Data *and* Safety and Monitoring Board (DSMC) notified within 5 business days of the PI become aware. Participants will not resume study drug use until the DSMC decides that the AE is not related to the study drug.

Outcomes of AEs will be assessed as: 1) Recovered, 2) Recovered with sequelae, 3) Ongoing at participant end-of-study visit, 4) Died, 5) Unknown.

Pregnancies detected during the study (after first use of Artesunate (Visit 1) through Visit 5)will be recorded as SAEs. The participant will be referred for prenatal care, preferably to a provider who is experienced in reproductive toxicity for further evaluation and counseling. With participant’s permission, any pregnancies occurring during study participation will be followed for pregnancy outcome. If permission is granted, the Investigator will follow the participant until completion of the pregnancy and document the outcome of the pregnancy (either normal or abnormal outcome) and report the condition of the fetus or newborn to the Study Coordinator, if possible. If the outcome of the pregnancy was abnormal (e.g., spontaneous or therapeutic abortion), the Investigator will report the abnormal outcome as an AE.

For all adverse events reported or observed, the information should be recorded in the Case Report Forms for that participant. This will include a full description of the event, its seriousness, its severity or toxicity grade (intensity), the relationship to the study drug, and the treatment, outcome, and sequelae of the event.

Any serious or unexpected event, which occurs to any participant in the course of her treatment on this study or within 30 days following cessation of treatment, must be reported immediately to the Study Coordinator by telephone at +254 705 755220 or within 24 hours of the investigator learning of its occurrence. The immediate reports should be followed promptly by detailed, written reports. The Study Coordinator will then inform the Principal Investigator of the Adverse Event The PI is responsible for reporting AEs.Copies of these reports should be sent to:

- - IRB
  - Funding Source (if appropriate)

Reporting is required of all unanticipated problems to the UNC IRB, including those which may occur after the participant has completed or has withdrawn from the study, including after study closure.

Unanticipated problems as defined by the UNC IRB:

Unanticipated problems involving risks to participants or others” or “Unanticipated Problem” (UP) refers to any incident, experience, or outcome that:

- is unexpected (in terms of nature, severity, or frequency) given (a) the research procedures that are described in the protocol-related documents, such as the IRB-approved research protocol and informed consent document; and (b) the characteristics of the participant population being studied;
- is related or possibly related to a participant’s participation in the research; and
- suggests that the research places participants or others at a greater risk of harm (including physical, psychological, economic, or social harm) related to the research than was previously known or recognized.

Unanticipated Problems that are serious adverse events should be reported to the UNC IRB within one (1) week of the investigator becoming aware of the event. Any other Unanticipated Problem should be reported to the UNC IRB within two (2) weeks of the investigator becoming aware of the problem. If the Unanticipated Problem Report cannot be completed in its entirety within the required time period, a preliminary report should be submitted. The Unanticipated Problem Report should be amended once the event is resolved and/or more information becomes available.

All individual AE and IND Safety Reports shall be maintained by the Principal Investigator. For those events that are not reportable to the UNC IRB, a summary (i.e., not individual reports) of all adverse events that have occurred within the last approval period should be submitted to the UNC and Kenya IRB’s at the time of continuing review.

Reports from a Data and Safety Monitoring Committee, (DSMC), or other independent safety monitoring group should be provided to the UNC and Kenya IRBs on a regular basis, generally at least as often as the study undergoes continuing review. Reports should include findings from adverse event reports and recommendations derived from data and safety monitoring.

## **5.4 Specimen collection, processing, storage and analysis**

For each participant, ~2.5 ml of blood will be collected at each timepoint at baseline (Day 1), and on day 5 prior to administration of intravaginal artesunate (time 0), and at 15, 30min, 1h, 2h, 4h, 6h and 8h after administration. Blood will be collected in gold/yellow top tubes and centrifuged to serum which will be stored at -80^0^C until analyzed. Samples will be stored at Kenya Medical Research Institute (KEMRI)-affiliated accredited laboratories in Kisumu, Kenya, with cold chain maintained from collection to storage until shipping for analysis. All study staff as well as laboratory staff will be trained on study SOPs.

**DHA and artesunate serum testing:** Specimens will be tested at **Strathmore University**, **Kenya** which has validated DHA and artesunate assays. Analysis will be led by Martin Ongas, Research Scientist at CREATES, Strathmore University. Serum samples will be shipped under cold chain and remain frozen at -80^0^C in Strathmore laboratory facilities until ready for analysis. Analysis will be performed using a validated high-performance Liquid Chromatography-tandem Mass Spectrometry (LC-MS/MS) assay, which has been optimized to enable simultaneous measurement of artesunate and DHA from the same sample aliquot. The limits of quantification of Artesunate and DHA at approximately 5ng/ml and 8ng/ml, respectively.^31,^ Quality control (QC) samples will be included with each assay batch, with the acceptance criteria requiring QC standards to be within +/- 15% of the nominal value. At least 67% of QC samples must pass at each concentration level (low, medium, high). ^35^ All appropriate material transfer agreements and shipping licenses will be obtained from all relevant regulatory bodies including the Amref ESRC before specimen shipping is done. All specimens will be destroyed following testing. Each sample will be assigned a unique code number, and no identifiable personal health information (PHI) will be on the specimen label.

**Pharmacokinetic analysis:** Conventional pharmacokinetic parameters - AUC (0, infinity), half-life, apparent clearance, the volume of distribution, maximum serum concentration (Cmax), time to Cmax will be determined from the serum concentration-time data using noncompartmental analysis.^32,35^ Peak serum concentration (Cmax) and time to reach peak concentration (tmax) for artesunate and DHA will be the observed values. The AUC of AS will be calculated by linear trapezoidal summation with extrapolation to infinity. With respect to DHA, AUC will be calculated to the last drug measurable time point. The elimination rate constant (β), will be calculated from the slope of the terminal phase of the log concentration-time profile, and the elimination half-life (t1/2) calculated from the ratio of ln 2/β. Other PK parameters will be calculated using standard model-independent formulae.^39^ The estimates of PK parameters for DHA will assume complete conversion of AS to DHA as reported previously.^35^ Data will be plotted graphically and analyzed using statistical software. Data will be presented as mean with standard deviation (SD).

**Compliance Statement:** Biospecimen collection for this study will be conducted in full accordance with all applicable University of North Carolina (UNC) Research Policies and Procedures including Good Clinical Practice (CGP) policies, and all applicable Federal and state laws and regulations including 45 CFR 46, and the Health Insurance Portability and Accountability Act Privacy Rule, as well as the in-country regulatory agencies including the Amref Ethics and Scientific Review Committee (ESRC), the Kenya Pharmacy and Poisons Board (PPB), the National Commission for Science, Technology & Innovation (NACOSTI) and any other regulatory bodies required in-country. Any episode of noncompliance will be documented. The investigators will perform the study in accordance with this protocol, will obtain consent, and will report unexpected problems in accordance with the UNC IRB Policies and Procedures and all federal requirements. Collection, recording, and reporting of data will be accurate and will ensure the privacy, health, and welfare of research participants during and after the study.

**Biosafety:** To ensure the safety of our research staff, study participants, and the surrounding community, we will follow the biosafety level 2 (BSL-2) standard as per the Kenyan Laboratory Biosafety and Biosecurity Policy Guidelines and Kenya National Guidelines for Safe Management of Health Care Waste. This standard is designed to reduce or eliminate exposure of laboratory workers, other persons, and the outside environment to potentially hazardous or detrimental materials. The study drug (artesunate) is a well-characterized drug that has been extensively studied and is not known to cause disease in healthy individuals. The biological material handled, and biological waste generated during the study are moderate-risk infectious agents and pose a moderate individual risk and low community risk of exposure. By following these guidelines, we can ensure that all our procedures are conducted safely, and that medical waste is disposed of properly.

**Monitoring/Data and Safety Monitoring:** The Principal Investigator will provide continuous monitoring of participant safety in this trial with periodic reporting to the Data and Safety Monitoring Committee (DSMC). Meetings/teleconferences will be held at a frequency dependent on study accrual. These meetings will include the investigators as well as study coordinators, data coordinators, regulatory associates, and any other relevant personnel the principal investigators may deem appropriate. At these meetings, the research team will discuss all issues relevant to study progress, including enrollment, safety, regulatory, data collection, etc. The team will produce summaries or minutes of these meetings. These summaries will be available for inspection when requested by any of the regulatory bodies charged with the safety of human participants and the integrity of data including, but not limited to, the oversight of Office of Human Research Ethics Biomedical IRB.

The UNC PI will be responsible for submitting the following information for review:

1. safety and accrual data including the number of participants treated;
2. significant developments reported in the literature that may affect the safety of participants or the ethics of the study;
3. preliminary response data; and
4. summaries of team meetings that have occurred since the last report. Findings of the DSMC review will be disseminated by memo to the UNC PI, the UNC IRB, and all Kenya regulatory bodies governing the study.

**Removal of Participants from the Study:** Any participant who does not wish to participate in the study will be exited from the study at any time they prefer. Additionally, participants who fail to adhere to significant protocol requirements, including failure to meet inclusion/exclusion criteria, use of prohibited concomitant medications, incident pregnancy, or other reasons that in the opinion of the Principal Investigator may affect participant safety may be removed from the study. The Investigator will determine if a protocol violation results in withdrawal of a participant. All protocol violations will be reported to the relevant governing IRBs.

Participants who permanently discontinue study product and follow-up prior to study completion will be asked to complete an early withdrawal visit, if willing, during which any AEs will be assessed. If study removal occurs due to pregnancy, participants will be referred to the nearest preferred prenatal facility for pregnancy care.

**Replacement of Participants:** A sample size of N=12 participants are needed for the primary aims of PK analysis. To allow for potential drop offs or incomplete evaluation, up to N=16 participants will be consented and enrolled to have a sample size of N=12 fully evaluable participants, who will finish all study visits and have all specimens collected. Once the minimum sample size of N=12 of fully eligible participants is met, no additional participants will be enrolled or initiate study procedures.

**Participant Withdrawal:** If a participant decides to withdraw from the study (and not just from protocol therapy) an effort should be made to complete and report study assessments as thoroughly as possible. At the time of withdrawal, the investigator should attempt to establish as completely as possible the reason for the study withdrawal.

- The participant should be asked if they are willing to allow for the abstraction of relevant information from their medical record in order to meet the objectives outlined in the protocol.
- A complete final evaluation at the time of the participant’s study withdrawal should be obtained with an explanation of why the participant is withdrawing from the study.
- If the participant is noncompliant and does not return for an end of study follow up assessment, this should be documented in the eCRF.
- If the reason for removal of a participant from the study is an adverse event, the principal specific event will be recorded on the eCRF.

Excessive participant withdrawals from protocol therapy or from the study can render the study uninterpretable; therefore, unnecessary withdrawal of participants should be avoided.

**Participant Lost to Follow up:** A participant will be considered lost to follow-up if he or she fails to return for three scheduled study visits and is unable to be contacted by the study site staff. The following actions must be taken if a participant fails to return to the clinic for a required study visit:

- The site will attempt to contact the participant and reschedule the missed visit and counsel the participant on the importance of maintaining the assigned visit schedule and ascertain if the participant wishes to and/or should continue in the study.
- Before a participant is deemed lost to follow-up, the investigator or designee will make every effort to regain contact with the participant (where possible, 3 telephone calls and, if necessary, a home visit by the study team if permission is obtained for home visit at study entry, or local equivalent methods). These contact attempts should be documented in the participant’s medical record or study file.
- Should the participant continue to be unreachable, he or she will be considered to have withdrawn from the study with a primary reason of lost to follow-up.

**Off-Study Criteria:** Participants will be considered off study and will no longer be followed on the protocol for any reason if any of the following criteria are met: when the participant reaches the end of the study as defined by the Schedule of Events Table, participant withdraws consent for treatment and any further data collection, death, or lost to follow-up.

**Malaria treatment during dosing phase (Day 1-5):** If study participants are diagnosed with malaria during the Artesunate pessary dosing phase (day 1-5), a non-ACT treatment will be used to avoid PK studies confounding. Participants will be encouraged to sleep under a mosquito net to reduce the chance of new malaria infection during the study.

##

## **5.5 Data Collection and Management**

**Data Collection and Management:** During study visits, a study nurse and/or a research assistant will collect demographic and clinical data for study use. Clinical data collected will include vital signs, medical history and examination, adverse events and other data as required. Data will be entered into REDCap EDC. A Study Analyst will review and clean study data regularly including performing quality assurance checks to ensure that there are not any aberrations in the data that require further clarification and/or changes to the study forms or database.

Variables to be collected will include basic demographic (e.g., age, education level, marital status) and clinical (e.g gravidity and parity, contraception use, pregnancy testing) variables as well as symptom review will be collected. Demographic and clinical characteristics will be used to describe the study population.

Study tools will include a baseline data collection and physical exam form; follow-up visit data collection forms (symptom and adherence evaluation and physical exam).

The UNC LCCC Data and Safety Monitoring Committee (DSMC) will review the study on a regular (quarterly to annually) basis, with the frequency of review based on risk and complexity as determined by the UNC Protocol Review Committee. The UNC PI will be responsible for submitting the following information for review: 1) safety and accrual data including the number of participants treated; 2) significant developments reported in the literature that may affect the safety of participants or the ethics of the study; 3) preliminary response data; and 4) summaries of team meetings that have occurred since the last report. Findings of the DSMC review will be disseminated by memo to the UNC PI, PRC, the UNC IRB, and DSMB, and all Kenya regulatory bodies governing the study.

**Data Storage:** Study records will be accessible only to study staff. The records will be held lockable cabinets at the study sites. Electronic data collected will be entered in REDCap EDC. The link between personal identity and unique identifier (PID) will be kept separate from study records and will not be included in the analytic database. This linkage will be destroyed at the conclusion of the study. REDCap EDC is compliant with clinical trial data management requirements.

## **5.6 Statistical Considerations and Data analysis**

### **5.6.1 Sample Size:**

A sample size of n=12 patients is needed for the primary objective of pharmacokinetics evaluation. To allow for potential drop offs, up to n=16 patients will be enrolled to have a sample size of n=12 fully evaluable participants.

**Sample Size Justification:** This is the first study to evaluate the PK disposition of artesunate after intravaginal administration and thus no prior data are available to base the sample size calculation. The proposed sample size is based on the standard number of patients evaluated in clinical PK studies, ^72–75^ and is adequate to perform pharmacokinetic serum parameters of artesunate and DHA in order to meet the study's primary objective.

**Accrual:** We anticipate enrolling 2 participants a week. Hence we expect to enroll 12-16 participants in 6- 8 weeks (1.5-2 months).

### **5.6.2 Data Analysis Plan**

**Primary Outcome:** serum concentration versus time curve (AUC) of dihydroartemisinin (DHA) following five consecutive days of self-administration of 200mg Artesunate vaginal inserts (pessaries) among healthy women in Kenya.

Primary Endpoint: Mean DHA AUC (ng*hr/ml) with standard deviation on Day 5 Statistical analysis: Mean DHA AUC will be compared to historical studies after IV, oral and rectal administration among adults following similar dosing. Results will be analyzed using the student’s t-test for one-sample observation and a p-value of 0.05 will be considered statistically significant.

**Secondary Outcomes**

1. Serum AUC of Artesunate following 5-day self-administration of 200mg Artesunate vaginal inserts (pessaries)
   1. Endpoint: Mean artesunate AUC (ng*hr/ml) with standard deviation on Day 5
   2. Statistical analysis: Mean artesunate AUC (ng*hr/ml) will be compared to historical studies after IV, oral and rectal administration among adults following similar dosing. Results will be analyzed using the student's t-test for one-sample observation and a p-value of 0.05 considered statistically significant.
2. Maximum concentration of Artesunate (Cmax) following 5-day self-administration of 200mg Artesunate vaginal inserts (pessaries)
   1. Endpoint: Mean artesunate Cmax (ng/ml) with standard deviation on Day 5
   2. Statistical analysis: Mean artesunate Cmax (ng/ml) will be compared to historical studies after IV, oral, and rectal administration among adults following similar dosing. Results will be analyzed using the student's t-test for one-sample observation, and a p-value of 0.05 considered statistically significant.
3. Maximum concentration of DHA (Cmax) following 5-day self-administration of 200mg Artesunate vaginal inserts (pessaries)
   1. Endpoint: Mean DHA Cmax (ng/ml) with standard deviation on Day 5
   2. Statistical analysis: Mean DHA Cmax (ng/ml) will be compared to historical studies after IV, oral and rectal administration among adults following similar dosing. Results will be analyzed using the student's t-test for one-sample observation and a p-value of 0.05 considered statistically significant.
4. Time to maximum concentration (Tmax) of Artesunate following 5-day self-administration of 200mg Artesunate vaginal inserts (pessaries)
   1. Endpoint: Mean Artesunate Tmax (mins) with standard deviation on Day 5
   2. Statistical analysis: Mean artesunate Tmax (mins) will be compared to historical studies after IV, oral, and rectal administration among adults following similar dosing. Results will be analyzed using the Mann-Whitney U-test for nonparametric data, and a p-value of 0.05 considered statistically significant.
5. Time to maximum concentration (Tmax) of DHA following 5-day self-administration of 200mg Artesunate vaginal inserts (pessaries)
   1. Endpoint: Mean DHA Tmax (mins) with standard deviation on Day 5
   2. Statistical analysis: Mean DHA Tmax (mins) will be compared to historical studies after IV, oral and rectal administration among adults following similar dosing. Results will be analyzed using the Mann-Whitney U-test for nonparametric data and a p-value of 0.05 considered statistically significant.
6. Artesunate half-life (t_1/2_) following 5-day self-administration of 200mg Artesunate vaginal inserts (pessaries)
   1. Endpoint: Mean Artesunate half-life (t_1/2_) (mins) with standard deviation on Day 5
   2. Statistical analysis: Mean Artesunate half-life (t_1/2_) (mins) will be compared to historical studies after IV, oral and rectal administration among adults following similar dosing. Results will be analyzed using the Mann-Whitney U-test for nonparametric data and a p-value of 0.05 considered statistically significant.
7. DHA half-life (t_1/2_) following 5-day self-administration of 200mg Artesunate vaginal inserts (pessaries)
   1. Endpoint: Mean DHA half-life (mins) with standard deviation on Day 5
   2. Statistical analysis: Mean DHA half-life (t_1/2_) (mins) will be compared to historical studies after IV, oral and rectal administration among adults following similar dosing. Results will be analyzed using the Mann-Whitney U-test for nonparametric data and a p-value of 0.05 considered statistically significant.
8. Apparent clearance of Artesunate (AS) following 5-day self-administration of 200mg Artesunate vaginal inserts (pessaries)
   1. Endpoint: Mean Artesunate clearance (L/Kg/hr) with standard deviation on Day 5
   2. Statistical analysis: Mean Artesunate clearance (L/Kg/hr) will be compared to historical studies after IV, oral and rectal administration among adults following similar dosing. Results will be analyzed using the students t-test for one-sample observation and a p-value of 0.05 considered statistically significant.
9. Apparent clearance of DHA following 5-day self-administration of 200mg Artesunate vaginal inserts (pessaries)
   1. Endpoint: Mean DHA clearance (L/Kg/hr) with standard deviation on Day
   2. Statistical analysis: Mean DHA clearance (L/Kg/hr) will be compared to historical studies after IV, oral and rectal administration among adults following similar dosing. Results will be analyzed using the students t-test for one-sample observation and a p-value of 0.05 considered statistically significant.
10. Volume of distribution of Artesunate (AS) following 5-day self-administration of 200mg Artesunate vaginal inserts (pessaries)
    1. Endpoint: Mean Artesunate volume of distribution (L/Kg) with standard deviation on Day 5
    2. Statistical analysis: Mean Artesunate volume of distribution (L/Kg) will be compared to historical studies after IV, oral and rectal administration among adults following similar dosing. Results will be analyzed using the students t-test for one-sample observation and a p-value of 0.05 considered statistically significant.
11. Volume of distribution of DHA following 5-day self-administration of 200mg Artesunate vaginal inserts (pessaries)
    1. Endpoint: Mean DHA volume of distribution (L/Kg) with standard deviation on Day 5
    2. Statistical analysis: Mean DHA volume of distribution (L/Kg) will be compared to historical studies after IV, oral and rectal administration among adults following similar dosing. Results will be analyzed using the students t-test for one-sample observation and a p-value of 0.05 considered statistically significant.
12. Safety of 5-day course of self-administrated 200mg Artesunate vaginal inserts (pessaries) among health women in Kenya
    1. Endpoint: Type, frequency, severity, and duration of reported and observed adverse events (AEs) using the U.S National Cancer Institute Common Terminology Criteria for Adverse Events, v5.0 (CTCAE 5.0) and the Division of AIDS Female Genital Adverse Events Grading Table
    2. Statistical Analysis: The counts of adverse events for each participant will be tabulated by severity (grades 1-5). The proportion of participants with a severe AE (grade 3 or higher) will be reported along the exact (Clopper-Pearson) one-sided upper 95% confidence bounds. The proportion of participants who report a severe AE (if any) will also be reported, along with an exact one-sided upper 95% confidence bound.
       1. The at-risk period for safety will begin at study visit 1 (first artesunate use) and will continue through study visit 6 (4-10 days after last artesunate use). Safety data will be reported for all participants who start artesunate use.

**Pharmacokinetic analysis:** Conventional pharmacokinetic parameters - AUC (0, infinity), half-life, apparent clearance, volume of distribution, maximum serum concentration (Cmax), time to Cmax will be determined from the serum concentration-time data using noncompartmental analysis.^35,76^ Peak serum concentration (Cmax) and time to reach peak concentration (Tmax) for artesunate and DHA will be the observed values. The AUC of AS will be calculated by linear trapezoidal summation with extrapolation to infinity, and AUC time curve will start from drug administration to the last quantifiable observation. All parameters will be calculated using time in hours after the first drug administration. With respect to DHA, AUC will be calculated to the last drug measurable time point. The elimination rate constant (beta) will be calculated from the slope of the terminal phase of the log concentration-time profile, and the elimination half-life (t1/2) calculated from the ratio of ln 2/beta. Other PK parameters will be calculated using standard model-independent formulae.^39^ The estimates of PK parameters for DHA will assume complete conversion of AS to DHA as reported previously.^35^ Data will be plotted graphically and analyzed using statistical software. Data will be presented as mean with standard deviation (SD).

## **5.7 Community Engagement Plan**

The target population will be sensitized about the study through talks in public forums including health facilities within Kisumu County where the study will be done.

## **5.8 Study Limitations, Risks and Mitigation of Risks**

The study will investigate the pharmacokinetics of artesunate following intravaginal use at the proposed dosing and frequency. Limitations include not evaluating pharmacodynamics or pharmacokinetics following different dosing. Risks of the study and mitigation of risks are discussed under Ethical Considerations.

## **5.9 Plans for Communicating Study Findings**

This study will provide important pharmacokinetics data to inform questions of serum levels if any following intravaginal artesunate use and implications for malaria resistance in endemic communities. The final study results will be shared with the study team, the study participants in lay language, the medical and scientific communities including all stakeholders, and the public. Results will be published in peer reviewed scientific journals, abstracts, posters and symposium presentations at conferences. Results will also be shared with relevant stakeholders including the Ministry of Health in Kenya.

# **6.0 ETHICAL CONSIDERATIONS**

All investigators and study staff will undergo online Good Clinical Practice (GCP) and Human Participants Protection (HSP) training to ensure high levels of adherence to ethical principles of clinical research. They will also be expected to meet basic professional training based on their roles and functions.

## **6.1 Institutional Review Board (IRB) Approval and Consent**

This study will be reviewed and approved by the IRB at the University of North Carolina (UNC), as well as all relevant regulatory bodies in Kenya, which include the Amref Ethics and Scientific Review Committee (ESRC), the Kenya Pharmacy and Poisons Board (PPB), the National Commission for Science, Technology & Innovation (NACOSTI) and any other regulatory bodies required in-country.

The Amref ESRC & UNC IRB’s will have the proper representation and function in accordance with federally mandated regulations. The IRB will approve the informed consent form (ICF), study protocol, any other written information to be provided to participants and other study related documents as per the ethics and regulatory submission requirements. All amendments (e.g., those that affect the conduct of the study or the safety of participants) will require approval from all regulatory bodies. Before the investigational product can be shipped to the investigational site, a shipping permit will be obtained from the Pharmacy and Poisons board.

In obtaining and documenting informed consent, the investigator will comply with the applicable regulatory requirement(s) and should adhere to Good Clinical Practice (GCP) and to ethical principles that have their origin in the Declaration of Helsinki.

Before recruitment and enrollment into this study, the patient will be given a full explanation of the study, including the goals of the study, the risks and benefits and will be given the opportunity to review the consent form. As part of informed consent, the voluntary nature of study participation will be emphasized to potential participants, including the fact that their participation, or non-participation in the study will not impact their ongoing or future care. Each consent form must include all the relevant elements currently required by the and local or state regulations. Once this essential information has been provided to the patient and the investigator is assured that the patient understands the implications of participating in the study, the patient will be asked to give consent to participate in the study by signing an IRB-approved consent form. The written informed consent form will be signed and personally dated by the patient and by the person who conducted the informed consent discussion. Informed consent will be obtained from all participants in their preferred languages. To compensate for participants time, including potential lost wages from time spent on study visits, each scheduled study visit will be reimbursed Kshs 1000 (approximately $10). Additionally, participants will be reimbursed for transport, taking into consideration average cost of travel to the clinic to support follow-up and retention, expected at approximately Kshs 500 ($5). On Visit # 5 when participants are expected to spend 6-8 hours in the study clinic, participants will be reimbursed an additional Kshs 2,500 (US $25) for lost wages. During study visits, participants may receive a meal or refreshment based on their waiting time.

## **6.2 Confidentiality and Privacy**

All efforts will be made to maintain participant confidentiality. This includes use of participant ID and not names in study documents, conducting all interviewing and consenting in private rooms. Study data will be entered into secure forms and tablets and uploaded to a secure server. Study records will be kept in locked cabinets and will be accessible only to study staff.

## **6.3 Potential Benefits**

Individual benefits to participants are minimal. All participants will undergo a physical examination at screening free of charge. The results of all tests will be communicated to all participants.

## **6.4 Potential Harms and Minimization of Harms**

Potential risks to participants include risk of adverse events (AEs) following artesunate pessary use, risks associated with venipuncture include slight pain during the entry of the needle into the skin, very rarely possible dizziness, and syncope, risk of breach of confidentiality from study participation, and theoretical (but minimal) risk of developing artemisinin partial resistance for malaria treatment following intravaginal use. The strategy to minimize these risks as discussed below:

**Risk of adverse events from artesunate pessary use:** Risk of adverse events are expected to be minimal based on the excellent safety profile of intravaginal artesunate at the planned dose and frequency in a U.S Phase I study. In that trial, of 37 drug-related AEs reported, 34 (92%) were grade 1 (mild), and only 3 (8%) were grade II, including 2 cases of vaginal yeast infection and 1 case of non-infective cystitis. In this study, there were no grade 3 or higher AEs and no intolerable side effects that led to study withdrawal. As such, we expect risks related to AEs in this trial to similarly be minimal. There are no data on safety of intravaginal artesunate in pregnancy. Pregnancy is an exclusion criterion in this study. All participants will have pregnancy testing done at study entry to rule out pregnancy and will be required to use contraception if of childbearing age during the 5-day dosing phase.

**Risk associated with venipuncture**: Participant monitoring, aseptic technique, including sterile disposable blood collection apparatus and adherence to standard medical precautions will reduce any risks associated with venipuncture to a minimum. The amount of blood to be taken for sampling will not be harmful to the participant’s health, as it is significantly less (<1/10^th^) the volume of a normal blood donation.

**Risk of developing artemisinin partial resistance for malaria treatment:** As discussed in section 3.1, the dosing used for intravaginal use is much lower than that used for the rectal artesunate formulation for malaria treatment. Compared to the rectum, vascularity at the vaginal and cervix is significantly less hence likelihood of systemic exposure is exceedingly low. As such, minimal to no appreciable levels of the active substance are expected following intravaginal use of artesunate at the planned dosing and frequency. The objective of this study is to confirm this.

**Risk of breach of confidentiality:** Other small risks associated with study participation include a risk of breach of confidentiality during the study. This will be minimized through using only a unique participant identification number, and not participant names or other identifiers. Study participation will include data collection regarding basic medical history. The risk of this is minimal as questions asked will be routine medical questions. However, all efforts will be made to reduce the risk of distress to participants including using experienced interviewers, offering interviews in privacy for all participants, and reviewing the confidentiality of all answers at the beginning of data collection. Study staff will monitor participants for any signs of psychological distress and refer participants for additional counseling, as needed.

# **7.0 STUDY MANAGEMENT AND ORGANIZATION**

## **7.1 Study Management**

**Required Documentation:** Before the study can be initiated at any site, the following documentation must be provided to the IGHID Office at the University of North Carolina.

- A copy of the official IRB approval letter for the protocol and informed consent(s)
- IRB membership list
- CVs and medical licensure for the principal investigator and any associate investigators who will be involved in the study.
- Form FDA 1572 appropriately filled out and signed with appropriate documentation (if required)
- A copy of the IRB-approved consent form(s)
- CLIA Laboratory certification and institution lab normal value
- Executed clinical research contract.

**Registration Procedures:** Participant enrollment will be tracked in the cloud-based electronic database which will be backed up daily. The PI and study coordinator will review accrual data weekly to ensure enrollment targets are met. Once the planned sample size is achieved, participant enrollment will stop, and the study will focus on follow-up visits.

**Adherence to the Protocol:** Except for an emergency situation in which proper care for the protection, safety, and well-being of the study patient requires alternative treatment, the study shall be conducted as described in the approved protocol. Protocols deviations or violations will be managed as per section titled Protocol Deviations.

**Emergency Modifications:** Investigators may implement a deviation from, or a change of, the protocol to eliminate an immediate hazard(s) to trial participants without prior Amref ESRC and UNC IRB approval. For any such emergency modification implemented, a UNC IRB modification form must be completed by UNC Research Personnel within five (5) business days of making the change. Similarly, for any emergency modification implemented, the Amref ESRC will be notified completed and communicated to the Amref ESRC within five (5) business days of making the change, or earlier as required by Amref ESRC.

**Other Protocol Deviations/Violations:** According to UNC’s IRB, a protocol deviation is any unplanned variance from an IRB approved protocol that:

- Is generally noted or recognized after it occurs
- Has no substantive effect on the risks to research participants
- Has no substantive effect on the scientific integrity of the research plan or the value of the data collected
- Did not result from willful or knowing misconduct on the part of the investigator(s).

An unplanned protocol variance is considered a violation if the variance meets any of the following criteria:

- Has harmed or increased the risk of harm to one or more research participants.
- Has damaged the scientific integrity of the data collected for the study.
- Results from willful or knowing misconduct on the part of the investigator(s).
- Demonstrates serious or continuing noncompliance with federal regulations, State laws, or University policies.

If a deviation or violation occurs, the following guidelines will be followed:

**Protocol Deviations:** Study personnel will record any deviations and report to any sponsor or data and safety monitoring committee in accordance with their policies. Deviations should be summarized and reported to the IRB at the same time of continuing review.

**Protocol Violations:** Violations should be reported by UNC personnel within one (1) week of the investigator becoming aware of the event using the same IRB online mechanism used to report UPIRSO.

**Study Discontinuation:** The study may be discontinued at any time by the ACTU, IRB, the NIAID, the pharmaceutical supporters, the FDA, the OHRP, or other government agencies as part of their duties to ensure that research participants are protected.

**Unanticipated Problems Involving Risks to Participants or Others (UPIRSO:** Any events that meet the criteria for “Unanticipated Problems” as defined by UNC’s IRB must be reported by the Study Coordinator using the IRB’s web-based reporting system.

**Amendments to the Protocol**: Should amendments to the protocol be required, the amendments will be originated and documented by the Principal Investigator at UNC. It should also be noted that when an amendment to the protocol substantially alters the study design or the potential risk to the patient, a revised consent form might be required.

The written amendment, and if required the amended consent form, must be sent to UNC’s IRB for approval prior to implementation.

**Record Retention**: Study documentation includes all Case Report Forms, data correction forms or queries, source documents, , monitoring logs/letters, and regulatory documents (e.g., protocol and amendments, IRB correspondence and approval, signed patient consent forms). Source documents include all recordings of observations or notations of clinical activities and all reports and records necessary for the evaluation and reconstruction of the clinical research study.

Government agency regulations and directives require that all study documentation pertaining to the conduct of a clinical trial must be retained by the study investigator. In the case of a study with a drug seeking regulatory approval and marketing, these documents shall be retained for at least two years after the last approval of marketing application in an International Conference on Harmonization (ICH) region. In all other cases, study documents should be kept on file until three years after the completion and final study report of this investigational study.

**Obligations of Investigators**: The Principal Investigator is responsible for the conduct of the clinical trial at the site in accordance with Title 21 of the Code of Federal Regulations and/or the Declaration of Helsinki. The Principal Investigator is responsible for personally overseeing the treatment of all study patients. The Principal Investigator must assure that all study site personnel, including sub-investigators and other study staff members, adhere to the study protocol and all FDA/GCP/NCI regulations and guidelines regarding clinical trials both during and after study completion. The Principal Investigator at each institution or site will be responsible for assuring that all the required data will be collected and entered onto the Case Report Forms (CRFs). Periodically, monitoring/auditing will be conducted by study personnel who will review participant records including consent forms, CRFs, and supporting data to ensure protection of participants. At the completion of the study, all case report forms will be reviewed by the Principal Investigator and will require his/her final signature to verify the accuracy of the data. The site investigator will make study documents (e.g., consent forms, drug distribution forms, CRFs) and pertinent hospital or clinic records readily available for inspection by the local IRB, the site monitors, the FDA, the NIAID, and the OHRP for confirmation of the study data.

## **7.2 Role of Investigators Principal Investigators and role of Principal Co-Investigators**

**Chemtai Mungo, MD, MPH**: Dr. Chemtai Mungo will lead protocol implementation including being lead in subject matter expertise, overseeing all aspects of protocol implementation and leading data analysis, interpretation, dissemination of results and future Phase II grant writing and implementation. Dr. Mungo or her designee (via delegation of duties log) will conduct study visits including physical exams and adverse event evaluations. All designee’s will be trained in all aspects of the study protocol and have valid Good Clinical Practice IRB certification.

**Jackton Omoto, MBChB, MMed:** Maseno University (Co-Principal Investigator): Dr. Omoto will lead protocol implementation and provide subject participant matter expertise, recruit, train and provide guidance on recruitment and retention to the study team, as well as provide support in building capacity of study providers to deliver the intervention. Dr. Omoto or his designee (via delegation of duties log) will conduct study visits including physical exams and adverse event evaluations. All designee’s will be trained in all aspects of the study protocol and have valid Good Clinical Practice IRB certification.

**Co-Investigators and Other Personnel and their roles**

**Cirillus Ogollah, BSc, DipCM,** KEMRI (Co-Investigator): Mr. Ogollah will support management of study field operations in Kenya including supervision and support of the study team, support the recruitment and enrollment strategies and support in dissemination of results.

**Greshon Rota, BPharm, KEMRI** (Study Pharmacist): Dr. Rota will participate in implementation of the study medicine and reporting of adverse effects. He will also act in liaison with the regulatory authorities to ensure study follows clinical trials rules and regulations.

**Felix Adundo, BSc** KEMRI (Biostatistician): Felix Adundo will be the study and analyst who will be responsible for data management, QA/QC, and will work with the clinical pharmacologist on pharmacokinetic study analysis.

**Brenda Misiko, MBChB** (Study Coordinator): Brenda Misiko will be the study coordinator responsible for study monitoring, quality control procedures, and more.

## **7.3 Study Timeline**

This study is expected to last approximately 3-4 months once activated. Anticipated timeline to activation is below.

- UNC & Kenya Ethics Review and approval: October 2023 – November 2023
- Kenya Poisons Board review and approval: December 2023 – February 2024
- Protocol training: January – February 2024
- Participant Accrual & Data collection: March 2024 – May 2024
- Data analysis and Dissemination: June 2024 - July 2024

## **7.4 Budget and Justification**

Kenya Budget: Total = US $40,000.00 (Approximately Kshs 5,200,000)

- Personnel (salaries): US $30,000 (Ksh 6,500,000)
- Personnel (fringe benefits): US $5,000 (Kshs 650,000)
- Other direct costs: US $ 5,000 (Kshs 650,000)

UNC: Total = $ 30,000

- Travel: $3,000
- Analysis: $ 27,000

**Budget Justification**

KEMRI: UNC will provide funds to KEMRI (sub-contract) to cover personnel salaries + fringe for research staff/consultants and study direct costs.

UNC: Funds will be available to UNC to facilitate PI travel to Kenya as well as for sample analysis.

# **8.0 REFERENCES**

1. Sung H, Ferlay J, Siegel RL, et al. Global Cancer Statistics 2020: GLOBOCAN Estimates of Incidence and Mortality Worldwide for 36 Cancers in 185 Countries. *CA Cancer J Clin*. 2021;71(3):209-249. doi:10.3322/caac.21660
2. Fonseca BO, Possati-Resende JC, Salcedo MP, et al. Topical Imiquimod for the Treatment of High-Grade Squamous Intraepithelial Lesions of the Cervix: A Randomized Controlled Trial. *Obstetrics and gynecology*. 2021;137(6):1043-1053. doi:10.1097/AOG.0000000000004384
3. Desravines N, Miele K, Carlson R, Chibwesha C, Rahangdale L. Topical therapies for the treatment of cervical intraepithelial neoplasia (CIN) 2-3: A narrative review. *Gynecol Oncol Rep*. 2020;33:100608. doi:10.1016/j.gore.2020.100608
4. Maiman M, Watts DH, Andersen J, Clax P, Merino M, Kendall MA. Vaginal 5-fluorouracil for high-grade cervical dysplasia in human immunodeficiency virus infection: A randomized trial. *Obstetrics and Gynecology*. 1999;94(6):954-961. doi:10.1016/S0029-7844(99)00407-X
5. de Witte CJ, van de Sande AJM, van Beekhuizen HJ, Koeneman MM, Kruse AJ, Gerestein CG. Imiquimod in cervical, vaginal and vulvar intraepithelial neoplasia: a review. *Gynecol Oncol*. 2015;139(2):377-384. doi:10.1016/j.ygyno.2015.08.018
6. Hendriks N, Koeneman MM, van de Sande AJM, et al. Topical Imiquimod Treatment of High-grade Cervical Intraepithelial Neoplasia (TOPIC-3): A Nonrandomized Multicenter Study. *J Immunother*. 2022;45(3):180-186. doi:10.1097/CJI.0000000000000414
7. World Health Organisation. *World Health Organization. Guidelines for Treatment of Malaria. 3rd Edition*.; 2015.
8. Bar-Zeev N, White N. Evidence behind the WHO guidelines: Hospital Care for Children: efficacy and safety of artemisinin derivatives in children with malaria. *J Trop Pediatr*. 2006;52(2):78-82. doi:10.1093/tropej/fml005
9. Johann-Liang R, Albrecht R. Safety evaluations of drugs containing artemisinin derivatives for the treatment of malaria. *Clin Infect Dis*. 2003;36(12):1626-1628. doi:10.1086/375089
10. Nyaaba N, Andoh NE, Amoh G, et al. Comparative efficacy and safety of the artemisinin derivatives compared to quinine for treating severe malaria in children and adults: A systematic update of literature and network meta-analysis. *PLoS One*. 2022;17(7):e0269391. doi:10.1371/journal.pone.0269391
11. Ellis T, Eze E, Raimi-Abraham BT. Malaria and Cancer: a critical review on the established associations and new perspectives. *Infect Agent Cancer*. 2021;16(1):33. doi:10.1186/s13027-021-00370-7
12. Ma Z, Woon CYN, Liu CG, et al. Repurposing Artemisinin and its Derivatives as Anticancer Drugs: A Chance or Challenge? *Front Pharmacol*. 2021;12:828856. doi:10.3389/fphar.2021.828856
13. Jeong DE, Song HJJ, Lim S, et al. Repurposing the anti-malarial drug artesunate as a novel therapeutic agent for metastatic renal cell carcinoma due to its attenuation of tumor growth, metastasis, and angiogenesis. *Oncotarget*. 2015;6(32):33046-33064. doi:10.18632/oncotarget.5422
14. Efferth T, Sauerbrey A, Olbrich A, et al. Molecular modes of action of artesunate in tumor cell lines. *Mol Pharmacol*. 2003;64(2):382-394. doi:10.1124/mol.64.2.382
15. Siegel EM, Patel N, Lu B, et al. Circulating biomarkers of iron storage and clearance of incident human papillomavirus infection. *Cancer Epidemiol Biomarkers Prev*. 2012;21(5):859-865. doi:10.1158/1055-9965.EPI-12-0073
16. Daniels TR, Bernabeu E, Rodríguez JA, et al. The transferrin receptor and the targeted delivery of therapeutic agents against cancer. *Biochim Biophys Acta*. 2012;1820(3):291-317. doi:10.1016/j.bbagen.2011.07.016
17. Schiffman M, Doorbar J, Wentzensen N, et al. Carcinogenic human papillomavirus infection. *Nat Rev Dis Primers*. 2016;2:16086. doi:10.1038/nrdp.2016.86
18. Trimble CL, Levinson K, Maldonado L, et al. A first-in-human proof-of-concept trial of intravaginal artesunate to treat cervical intraepithelial neoplasia 2/3 (CIN2/3). *Gynecol Oncol*. 2020;157(1):188-194. doi:10.1016/j.ygyno.2019.12.035
19. Wong YK, Xu C, Kalesh KA, et al. Artemisinin as an anticancer drug: Recent advances in target profiling and mechanisms of action. *Med Res Rev*. 2017;37(6):1492-1517. doi:10.1002/med.21446
20. Zhou X, Chen Y, Wang F, et al. Artesunate induces autophagy dependent apoptosis through upregulating ROS and activating AMPK-mTOR-ULK1 axis in human bladder cancer cells. *Chem Biol Interact*. 2020;331:109273. doi:10.1016/j.cbi.2020.109273
21. Zhang J, Zhou L, Xiang JD, Jin CS, Li MQ, He YY. Artesunate-induced ATG5-related autophagy enhances the cytotoxicity of NK92 cells on endometrial cancer cells via interactions between CD155 and CD226/TIGIT. *Int Immunopharmacol*. 2021;97:107705. doi:10.1016/j.intimp.2021.107705
22. Yao X, Zhao CR, Yin H, Wang K, Gao JJ. Synergistic antitumor activity of sorafenib and artesunate in hepatocellular carcinoma cells. *Acta Pharmacol Sin*. 2020;41(12):1609-1620. doi:10.1038/s41401-020-0395-5
23. Gao F, Sun Z, Kong F, Xiao J. Artemisinin-derived hybrids and their anticancer activity. *Eur J Med Chem*. 2020;188:112044. doi:10.1016/j.ejmech.2020.112044
24. Guan X, Guan Y. Artemisinin induces selective and potent anticancer effects in drug resistant breast cancer cells by inducing cellular apoptosis and autophagy and G2/M cell cycle arrest. *J BUON*. 2020;25(3):1330-1336.
25. Zhang B. Artemisinin-derived dimers as potential anticancer agents: Current developments, action mechanisms, and structure-activity relationships. *Arch Pharm (Weinheim)*. 2020;353(2):e1900240. doi:10.1002/ardp.201900240
26. Li D, Zhang J, Zhao X. Mechanisms and Molecular Targets of Artemisinin in Cancer Treatment. *Cancer Invest*. 2021;39(8):675-684. doi:10.1080/07357907.2021.1954190
27. Anfosso L, Efferth T, Albini A, Pfeffer U. Microarray expression profiles of angiogenesis-related genes predict tumor cell response to artemisinins. *Pharmacogenomics J*. 2006;6(4):269-278. doi:10.1038/sj.tpj.6500371
28. Disbrow GL, Baege AC, Kierpiec KA, et al. Dihydroartemisinin is cytotoxic to papillomavirus-expressing epithelial cells in vitro and in vivo. *Cancer Res*. 2005;65(23):10854-10861. doi:10.1158/0008-5472.CAN-05-1216
29. Morris CA, Duparc S, Borghini-Fuhrer I, Jung D, Shin CS, Fleckenstein L. Review of the clinical pharmacokinetics of artesunate and its active metabolite dihydroartemisinin following intravenous, intramuscular, oral or rectal administration. *Malar J*. 2011;10:263. doi:10.1186/1475-2875-10-263
30. Ilett KF, Batty KT, Powell SM, et al. The pharmacokinetic properties of intramuscular artesunate and rectal dihydroartemisinin in uncomplicated falciparum malaria. *Br J Clin Pharmacol*. 2002;53(1):23-30. doi:10.1046/j.0306-5251.2001.01519.x
31. Ilett KF, Batty KT, Powell SM, et al. The pharmacokinetic properties of intramuscular artesunate and rectal dihydroartemisinin in uncomplicated falciparum malaria. *Br J Clin Pharmacol*. 2002;53(1):23-30. doi:10.1046/j.0306-5251.2001.01519.x
32. KF I, KT B, SM P, et al. The pharmacokinetic properties of intramuscular artesunate and rectal dihydroartemisinin in uncomplicated falciparum malaria. *Br J Clin Pharmacol*. 2002;53(1):23-30. doi:10.1046/J.0306-5251.2001.01519.X
33. Morris CA, Duparc S, Borghini-Fuhrer I, Jung D, Shin CS, Fleckenstein L. Review of the clinical pharmacokinetics of artesunate and its active metabolite dihydroartemisinin following intravenous, intramuscular, oral or rectal administration. *Malar J*. 2011;10:263. doi:10.1186/1475-2875-10-263
34. Batty KT, Thu LT, Davis TM, et al. A pharmacokinetic and pharmacodynamic study of intravenous vs oral artesunate in uncomplicated falciparum malaria. *Br J Clin Pharmacol*. 1998;45(2):123-129. doi:10.1046/j.1365-2125.1998.00655.x
35. Hien TT, Davis TME, Chuong L V., et al. Comparative pharmacokinetics of intramuscular artesunate and artemether in patients with severe falciparum malaria. *Antimicrob Agents Chemother*. 2004;48(11):4234-4239. doi:10.1128/AAC.48.11.4234-4239.2004
36. Diem Thuy LT, Ngoc Hung L, Danh PT, Na-Bangchang K. Absence of time-dependent artesunate pharmacokinetics in healthy subjects during 5-day oral administration. *Eur J Clin Pharmacol*. 2008;64(10):993-998. doi:10.1007/s00228-008-0506-6
37. Diem Thuy LT, Ngoc Hung L, Danh PT, Na-Bangchang K. Absence of time-dependent artesunate pharmacokinetics in healthy subjects during 5-day oral administration. *Eur J Clin Pharmacol*. 2008;64(10):993-998. doi:10.1007/S00228-008-0506-6
38. Krudsood S, Looareesuwan S, Tangpukdee N, et al. New fixed-dose artesunate-mefloquine formulation against multidrug-resistant Plasmodium falciparum in adults: a comparative phase IIb safety and pharmacokinetic study with standard-dose nonfixed artesunate plus mefloquine. *Antimicrob Agents Chemother*. 2010;54(9):3730-3737. doi:10.1128/AAC.01187-09
39. Navaratnam V, Mansor SM, Mordi MN, Akbar A, Abdullah MN. Comparative pharmacokinetic study of oral and rectal formulations of artesunic acid in healthy volunteers. *Eur J Clin Pharmacol*. 1998;54(5):411-414. doi:10.1007/S002280050484
40. Sirivichayakul C, Sabchareon A, Pengsaa K, et al. Comparative study of the effectiveness and pharmacokinetics of two rectal artesunate/oral mefloquine combination regimens for the treatment of uncomplicated childhood falciparum malaria. *Ann Trop Paediatr*. 2007;27(1):17-24. doi:10.1179/146532807X170466
41. Halpaap B, Ndjave M, Paris M, Benakis A, Kremsner PG. Plasma levels of artesunate and dihydroartemisinin in children with Plasmodium falciparum malaria in Gabon after administration of 50-milligram artesunate suppositories. *Am J Trop Med Hyg*. 1998;58(3):365-368. doi:10.4269/ajtmh.1998.58.365
42. Navaratnam V, Mansor SM, Mordi MN, Akbar A, Abdullah MN. Comparative pharmacokinetic study of oral and rectal formulations of artesunic acid in healthy volunteers. *Eur J Clin Pharmacol*. 1998;54(5):411-414. doi:10.1007/s002280050484
43. Teja-isavadharm P, Watt G, Eamsila C, et al. Comparative pharmacokinetics and effect kinetics of orally administered artesunate in healthy volunteers and patients with uncomplicated falciparum malaria. *Am J Trop Med Hyg*. 2001;65(6):717-721. doi:10.4269/AJTMH.2001.65.717
44. Binh TQ, Ilett KF, Batty KT, et al. Oral bioavailability of dihydroartemisinin in Vietnamese volunteers and in patients with falciparum malaria. *Br J Clin Pharmacol*. 2001;51(6):541-546. doi:10.1046/J.1365-2125.2001.01395.X
45. Guidelines for the Treatment of Malaria - PubMed. Accessed October 22, 2023. https://pubmed.ncbi.nlm.nih.gov/26020088/
46. Ward KE, Fidock DA, Bridgford JL. Plasmodium falciparum resistance to artemisinin-based combination therapies. *Curr Opin Microbiol*. 2022;69:102193. doi:10.1016/j.mib.2022.102193
47. Hanboonkunupakarn B, Tarning J, Pukrittayakamee S, Chotivanich K. Artemisinin resistance and malaria elimination: Where are we now? *Front Pharmacol*. 2022;13. doi:10.3389/FPHAR.2022.876282
48. Balikagala B, Fukuda N, Ikeda M, et al. Evidence of Artemisinin-Resistant Malaria in Africa. *N Engl J Med*. 2021;385(13):1163-1171. doi:10.1056/NEJMoa2101746
49. Mathenge PG, Low SK, Vuong NL, et al. Efficacy and resistance of different artemisinin-based combination therapies: a systematic review and network meta-analysis. *Parasitol Int*. 2020;74. doi:10.1016/J.PARINT.2019.04.016
50. Tilley L, Straimer J, Gnädig NF, Ralph SA, Fidock DA. Artemisinin Action and Resistance in Plasmodium falciparum. *Trends Parasitol*. 2016;32(9):682-696. doi:10.1016/J.PT.2016.05.010
51. Siddiqui FA, Liang X, Cui L. Plasmodium falciparum resistance to ACTs: Emergence, mechanisms, and outlook. *Int J Parasitol Drugs Drug Resist*. 2021;16:102-118. doi:10.1016/J.IJPDDR.2021.05.007
52. Mok S, Stokes BH, Gnädig NF, et al. Artemisinin-resistant K13 mutations rewire Plasmodium falciparum’s intra-erythrocytic metabolic program to enhance survival. *Nat Commun*. 2021;12(1). doi:10.1038/S41467-020-20805-W
53. Witkowski B, Amaratunga C, Khim N, et al. Novel phenotypic assays for the detection of artemisinin-resistant Plasmodium falciparum malaria in Cambodia: in-vitro and ex-vivo drug-response studies. *Lancet Infect Dis*. 2013;13(12):1043-1049. doi:10.1016/S1473-3099(13)70252-4
54. Ward KE, Fidock DA, Bridgford JL. Plasmodium falciparum resistance to artemisinin-based combination therapies. *Curr Opin Microbiol*. 2022;69. doi:10.1016/J.MIB.2022.102193
55. Ndwiga L, Kimenyi KM, Wamae K, et al. A review of the frequencies of Plasmodium falciparum Kelch 13 artemisinin resistance mutations in Africa. *Int J Parasitol Drugs Drug Resist*. 2021;16:155-161. doi:10.1016/J.IJPDDR.2021.06.001
56. Moser KA, Madebe RA, Aydemir O, et al. Describing the current status of Plasmodium falciparum population structure and drug resistance within mainland Tanzania using molecular inversion probes. *Mol Ecol*. 2021;30(1):100-113. doi:10.1111/MEC.15706
57. Asua V, Conrad MD, Aydemir O, et al. Changing Prevalence of Potential Mediators of Aminoquinoline, Antifolate, and Artemisinin Resistance Across Uganda. *J Infect Dis*. 2021;223(6):985-994. doi:10.1093/INFDIS/JIAA687
58. Squier CA, Mantz MJ, Schlievert PM, Davis CC. Porcine vagina ex vivo as a model for studying permeability and pathogenesis in mucosa. *J Pharm Sci*. 2008;97(1):9-21. doi:10.1002/jps.21077
59. Meshnick SR. Artemisinin: mechanisms of action, resistance and toxicity. *Int J Parasitol*. 2002;32(13):1655-1660. doi:10.1016/s0020-7519(02)00194-7
60. Efferth T, Kaina B. Toxicity of the antimalarial artemisinin and its dervatives. *Crit Rev Toxicol*. 2010;40(5):405-421. doi:10.3109/10408441003610571
61. Zhao Y. Studies on systemic pharmacological effects of artesunate. *J Trop Med Hyg*. 1985;88(6):391-396.
62. Miller RS, Li Q, Cantilena LR, et al. Pharmacokinetic profiles of artesunate following multiple intravenous doses of 2, 4, and 8 mg/kg in healthy volunteers: phase 1b study. *Malar J*. 2012;11:255. doi:10.1186/1475-2875-11-255
63. Batty KT, Thu LT, Davis TM, et al. A pharmacokinetic and pharmacodynamic study of intravenous vs oral artesunate in uncomplicated falciparum malaria. *Br J Clin Pharmacol*. 1998;45(2):123-129. doi:10.1046/j.1365-2125.1998.00655.x
64. Krishna S, Planche T, Agbenyega T, et al. Bioavailability and preliminary clinical efficacy of intrarectal artesunate in Ghanaian children with moderate malaria. *Antimicrob Agents Chemother*. 2001;45(2):509-516. doi:10.1128/AAC.45.2.509-516.2001
65. Navaratnam V, Mansor SM, Sit NW, Grace J, Li Q, Olliaro P. Pharmacokinetics of artemisinin-type compounds. *Clin Pharmacokinet*. 2000;39(4):255-270. doi:10.2165/00003088-200039040-00002
66. Presnell AL, Chuchuen O, Simons MG, Maher JR, Katz DF. Full depth measurement of tenofovir transport in rectal mucosa using confocal Raman spectroscopy and optical coherence tomography. *Drug Deliv Transl Res*. 2018;8(3):843-852. doi:10.1007/s13346-018-0495-7
67. Global Malaria Programme WHO. *Rectal Artesunate for Pre-Referral Treatment of Severe Malaria*.; 2017.
68. Gomes M, Ribeiro I, Warsame M, Karunajeewa H, Petzold M. Rectal artemisinins for malaria: a review of efficacy and safety from individual patient data in clinical studies. *BMC Infect Dis*. 2008;8:39. doi:10.1186/1471-2334-8-39
69. U.S National Cancer Institute. *Common Terminology Criteria for Adverse Events (CTCAE) Version 5.0*.; 2017.
70. NIAID NIH. Division of AIDS Table for Grading the Severity of Adult and Pediatric Adverse Events. Accessed July 27, 2020. https://rsc.niaid.nih.gov/sites/default/files/addendum-1-female-genital-grading-table-v1-n ov-2007.pdf
71. Batty KT, Davis TME, Thu LTA, Binh TQ, Anh TK, Ilett KF. Selective high-performance liquid chromatographic determination of artesunate and alpha- and beta-dihydroartemisinin in patients with falciparum malaria. *J Chromatogr B Biomed Appl*. 1996;677(2):345-350. doi:10.1016/0378-4347(95)00428-9
72. Piha-Paul SA, Thein KZ, De Souza P, et al. First-in-human, phase I/IIa study of CRLX301, a nanoparticle drug conjugate containing docetaxel, in patients with advanced or metastatic solid malignancies. *Invest New Drugs*. 2021;39(4):1047-1056. doi:10.1007/s10637-021-01081-x
73. Ogungbenro K, Aarons L. How many subjects are necessary for population pharmacokinetic experiments? Confidence interval approach. *Eur J Clin Pharmacol*. 2008;64(7):705-713. doi:10.1007/s00228-008-0493-7
74. Zamboni WC, Combest AJ, DeLoia JA, et al. Pharmacologic and phenotypic study of docetaxel in patients with ovarian or primary peritoneal cancer. *Cancer Chemother Pharmacol*. 2011;68(5):1255-1262. doi:10.1007/s00280-011-1609-9
75. Zamboni WC, Ramalingam S, Friedland DM, et al. Phase I and pharmacokinetic study of pegylated liposomal CKD-602 in patients with advanced malignancies. *Clin Cancer Res*. 2009;15(4):1466-1472. doi:10.1158/1078-0432.CCR-08-1405
76. Bethell DB, Teja-Isavadharm P, Cao XT, et al. Pharmacokinetics of oral artesunate in children with moderately severe Plasmodium falciparum malaria. *Trans R Soc Trop Med Hyg*. 1997;91(2):195-198. doi:10.1016/s0035-9203(97)90222-4

# **9.0 Appendices**

1. Consent Form
2. U.S National Cancer Institute Common Terminology Criteria for Adverse Events, v5.0
3. Investigational Drug Accountability Record
4. Investigational Brochure
5. Participant Study Card
6. Participant Self-Administration Pictorial Guide (English, Swahili, Luo)

L. Division of AIDS Female Genital Adverse Events Grading Table M: Packaging of the Investigational Product

N: Delegation of Duty Log
